# GOLPH3 Is Essential for Contractile Ring Formation and Rab11 Localization to the Cleavage Site during Cytokinesis in *Drosophila melanogaster*

**DOI:** 10.1371/journal.pgen.1004305

**Published:** 2014-05-01

**Authors:** Stefano Sechi, Gianni Colotti, Giorgio Belloni, Vincenzo Mattei, Anna Frappaolo, Grazia D. Raffa, Margaret T. Fuller, Maria Grazia Giansanti

**Affiliations:** 1 Istituto di Biologia e Patologia Molecolari del CNR, Dipartimento di Biologia e Biotecnologie, Università Sapienza di Roma, Roma, Italy; 2 Istituto di Biologia e Patologia Molecolari del CNR, Dipartimento di Scienze Biochimiche, Università di Roma Sapienza, Roma, Italy; 3 Laboratory of Experimental and Environmental Pathology, Sabina Universitas, Rieti, Italy; 4 Dipartimento di Biologia e Biotecnologie, Università Sapienza di Roma, Roma, Italy; 5 Departments of Developmental Biology and Genetics, Stanford University School of Medicine, Stanford, California, United States of America; University of Cambridge, United Kingdom

## Abstract

The highly conserved Golgi phosphoprotein 3 (GOLPH3) protein has been described as a Phosphatidylinositol 4-phosphate [PI(4)P] effector at the Golgi. GOLPH3 is also known as a potent oncogene, commonly amplified in several human tumors. However, the molecular pathways through which the oncoprotein GOLPH3 acts in malignant transformation are largely unknown. GOLPH3 has never been involved in cytokinesis. Here, we characterize the *Drosophila melanogaster* homologue of human GOLPH3 during cell division. We show that GOLPH3 accumulates at the cleavage furrow and is required for successful cytokinesis in *Drosophila* spermatocytes and larval neuroblasts. In premeiotic spermatocytes GOLPH3 protein is required for maintaining the organization of Golgi stacks. In dividing spermatocytes GOLPH3 is essential for both contractile ring and central spindle formation during cytokinesis. Wild type function of GOLPH3 enables maintenance of centralspindlin and Rho1 at cell equator and stabilization of Myosin II and Septin rings. We demonstrate that the molecular mechanism underlying GOLPH3 function in cytokinesis is strictly dependent on the ability of this protein to interact with PI(4)P. Mutations that abolish PI(4)P binding impair recruitment of GOLPH3 to both the Golgi and the cleavage furrow. Moreover telophase cells from mutants with defective GOLPH3-PI(4)P interaction fail to accumulate PI(4)P-and Rab11-associated secretory organelles at the cleavage site. Finally, we show that GOLPH3 protein interacts with components of both cytokinesis and membrane trafficking machineries in *Drosophila* cells. Based on these results we propose that GOLPH3 acts as a key molecule to coordinate phosphoinositide signaling with actomyosin dynamics and vesicle trafficking during cytokinesis. Because cytokinesis failures have been associated with premalignant disease and cancer, our studies suggest novel insight into molecular circuits involving the oncogene *GOLPH3* in cytokinesis.

## Author Summary

In animal cell cytokinesis, constriction of an actomyosin ring at the equatorial cortex of dividing cells must be finely coordinated with plasma membrane remodeling and vesicle trafficking at the cleavage furrow. Accurate control of these events during cell cleavage is essential for maintaining ploidy and preventing neoplastic transformation. GOLPH3 has been recognized as a potent oncogene, involved in the development of several human tumors. However, the precise roles played by GOLPH3 in tumorigenesis are not yet understood. In this manuscript we demonstrate for the first time the requirement for GOLPH3 for cytokinesis. GOLPH3 protein localizes at the cleavage site of *Drosophila* dividing cells and is essential for cytokinesis in male meiotic cells and larval neuroblasts. We show that this protein acts as a key molecule in coupling plasma membrane remodeling with actomyosin ring assembly and stability during cytokinesis. Our studies indicate a novel connection between *GOLPH3* and the molecular mechanisms of cytokinesis, opening new fields of investigation into the tumor cell biology of this oncogene.

## Introduction

Cytokinesis in animal cells is achieved through the constriction of a plasma membrane-anchored actomyosin ring that drives cleavage furrow ingression from the equatorial cortex [1]. A network of cytoskeletal proteins, including Septins and Anillin, acts as a scaffold to anchor the actomyosin ring to the plasma membrane during furrowing [2], [3].

The central spindle (the prominent microtubule bundle that forms in ana-telophase between the segregating chromosomes) dictates the position of the cleavage furrow [4]–[6]. Central spindle assembly is regulated by the coordinated action of kinesin motor proteins, microtubule associated proteins (MAPs) and protein kinases [6]. A crucial signaling event that sets up the site of cleavage furrow formation for cytokinesis is the accumulation of active Rho GTPase at an equatorial position at the cortex. This localized active Rho GTPase drives both F-actin assembly at the plasma membrane and Myosin II activation [4], [5]. The central spindle transmits the spatial information required for cleavage furrow formation by delivering regulators of Rho to the equatorial cortex [4], [6].

Recent studies have shown that besides contractile ring constriction, cytokinesis also involves membrane trafficking from internal stores to the cleavage furrow [7], [8]. Work from several groups has implicated Golgi-derived vesicles in cytokinesis [7]–[9]. Endocytic traffic also contributes to this process. Clathrin and Dynamin, two proteins that promote endocytic vesicle budding from the plasma membrane and the endocytic recycling factors ARF6, Rab35 and Rab11 are required for completion of cytokinesis in several cell systems [7]–[9].

Successful cytokinesis also depends on a special plasma membrane lipid composition [9]–[11]. It has been suggested that special lipids at the cleavage site facilitate cell shape remodeling during furrow ingression, regulate membrane addition and/or provide signalling platforms controlling cytokinesis [8]. However the molecular mechanisms underlying membrane-cytoskeleton interactions during cleavage furrow ingression are not yet understood.

Several proteins involved in the phosphoinositide (PIP) cycle and specific types of PIPs have been implicated in cytokinesis. For example the *Drosophila melanogaster* phosphatidylinositol transfer protein Giotto/Vibrator (Gio/Vib) and the Golgi PI 4-kinase Four wheel drive (Fwd) are both required for furrow ingression in male meiotic cytokinesis [12]–[15]. Fwd is required to recruit Rab11 at the Golgi, for the synthesis of PI(4)P on Golgi membranes and for accumulation of Rab11- and PI(4)P-containing organelles at the cell equator [15]. However Fwd does not accumulate at the cleavage furrow during cytokinesis [15].

GOLPH3, a highly conserved 34-kDa protein, functions as a PI(4)P effector at the Golgi [16], [17]. Recruitment of GOLPH3 and its yeast orthologue Vps74p to the Golgi depends on specific recognition of PI(4)P through a conserved binding pocket on the surface of the protein [16], [17]. Human GOLPH3 also interacts with MYO18A, mediating linkage with the F-actin cytoskeleton that has been proposed to facilitate flattening of the Golgi and vesicle formation [16]. GOLPH3 is also a potent oncogene, as it is commonly amplified in several solid tumours [18]–[27]. However, the molecular mechanisms through which this protein acts in malignant transformation are not yet understood. GOLPH3 has been implicated in cellular transformation via changes in the activity of the mammalian target of rapamycin (mTOR) [18]. GOLPH3 has been shown to activate mTOR signaling [18]. In addition GOLPH3 protein physically interacts with the VPS35 subunit of the retromer protein-recycling complex which has been linked to TOR signaling in yeast [18].

Here we present the first evidence to date implicating GOLPH3 in cytokinesis. We demonstrate that the *Drosophila* homologue of GOLPH3 controls both contractile ring formation and vesicle trafficking during cleavage furrow ingression in dividing cells. In addition, this protein accumulates at the midzone of telophase cells and interacts with components of both the cytokinetic apparatus and the membrane trafficking machinery. We propose that during cell cleavage, GOLPH3 acts as a key molecule to coordinate membrane remodeling and cytoskeletal dynamics.

## Results

### Isolation of mutants in *Drosophila GOLPH3*

**Figure 1:**
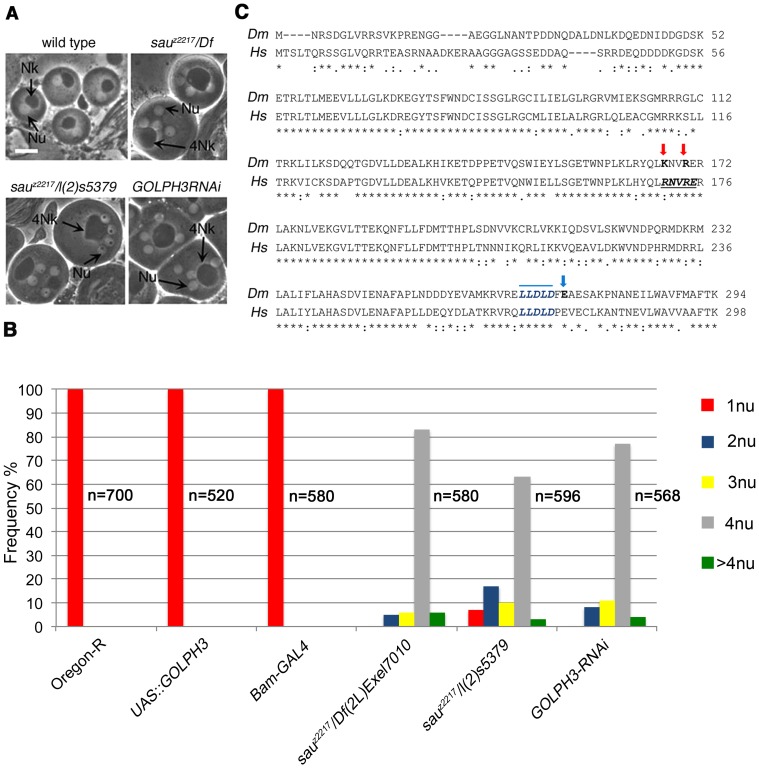
*Drosophila* GOLPH3 is required for spermatocyte cytokinesis. (A) Multinucleate onion stage spermatids due to cytokinesis failure during meiosis in *GOLPH3* mutant males [*sau^z2217^/Df(2L)Exel7010* and *sau^z2217^/l(2)5379*] and in males expressing dsRNA against *CG7085* (*GOLPH3RNAi*), under control of the *Bam-GAL4* driver [74]. (Nu) nuclei; (Nk) mitochondrial derivative; (4Nk) large mitochondrial derivative. Scale Bar; 10 µm. (B) Frequencies of spermatids containing 1, 2, 3, 4 or more than 4 nuclei per Nk, in *sau^z2217^/Df(2L)Exel7010* and *sau^z2217^/l(2)5379* mutant males and in males expressing dsRNA against *CG7085* (*GOLPH3RNAi*) under control of *Bam-GAL4*. Oregon R males and males carrying either the *Bam-GAL4* driver alone or the *UAS::GOLPH3RNAi* construct alone were used as control. (n); total number of spermatids (C) Alignment of *Drosophila* GOLPH3 (*Dm*) protein with human GOLPH3 (*Hs*). (Blue arrowhead) substitution of Glutamic acid by Lysine (E273K) in the *sau^z2217^* male sterile allele; (red arrows) K167A and R170A mutations; (blue line) clathrin box sequence. (*) fully conserved residue; (**:**) conservation between groups of strongly similar properties; (**.**) conservation between groups of weakly similar properties.

The *sauron^z2217^ (sau^z2217^)* allele was identified in a screen for mutations that cause failure of cytokinesis in *Drosophila* spermatocytes [28]. The *sau^z2217^* mutation failed to complement both *Df(2L)Exel6007* and *Df(2L)Exel7010* for the male sterility and male meiotic defects, defining a small chromosome region containing 21 genes. A P-element insertion in the annotated *CG7085* gene, resulting in the lethal mutation *l(2)s5379*, failed to complement *sau^z2217^* for both the male sterility and meiotic cytokinesis phenotype (Figure 1A and 1B). In addition, testes from males expressing double stranded RNA (dsRNA) against *CG7085* in meiotic cells, contained frequent aberrant multi-nucleate spermatids, indicating cytokinesis defects comparable to those observed in *sau^z2217^*/*l(2)s5379* or *sau* ***^z2217^***/*Df(2L)Exel7010 (sau* ***^z2217^*** */Df)* males (Figure 1A and 1B). *CG7085* encodes a polypeptide of 293 amino acids that is 70% identical to human GOLPH3. Thus, hereafter we refer to *CG7085* as *Drosophila GOLPH3*. DNA sequencing of *sau^z2217^* allele (See Materials and Methods), revealed a single point mutation in the *GOLPH3* gene, resulting in the replacement of the conserved Glutamic-acid 273 by Lysine at the C-terminus of the predicted polypeptide (Figure 1C). Based on these results, *sau^z2217^* is a missense allele of the *Drosophila* homologue of GOLPH3 (Figure 1C). GFP-GOLPH3 protein expressed under the control of a tubulin promoter rescued the defects of *sau* ***^z2217^***/*Df* and *sau^z2217^*/*l(2)s5379* mutants, confirming that the cytokinesis phenotype is the consequence of alterations in the *Drosophila* GOLPH3 homologue.

### The *Drosophila* homologue of mammalian GOLPH3 localized to the cleavage site in dividing spermatocytes

**Figure 2:**
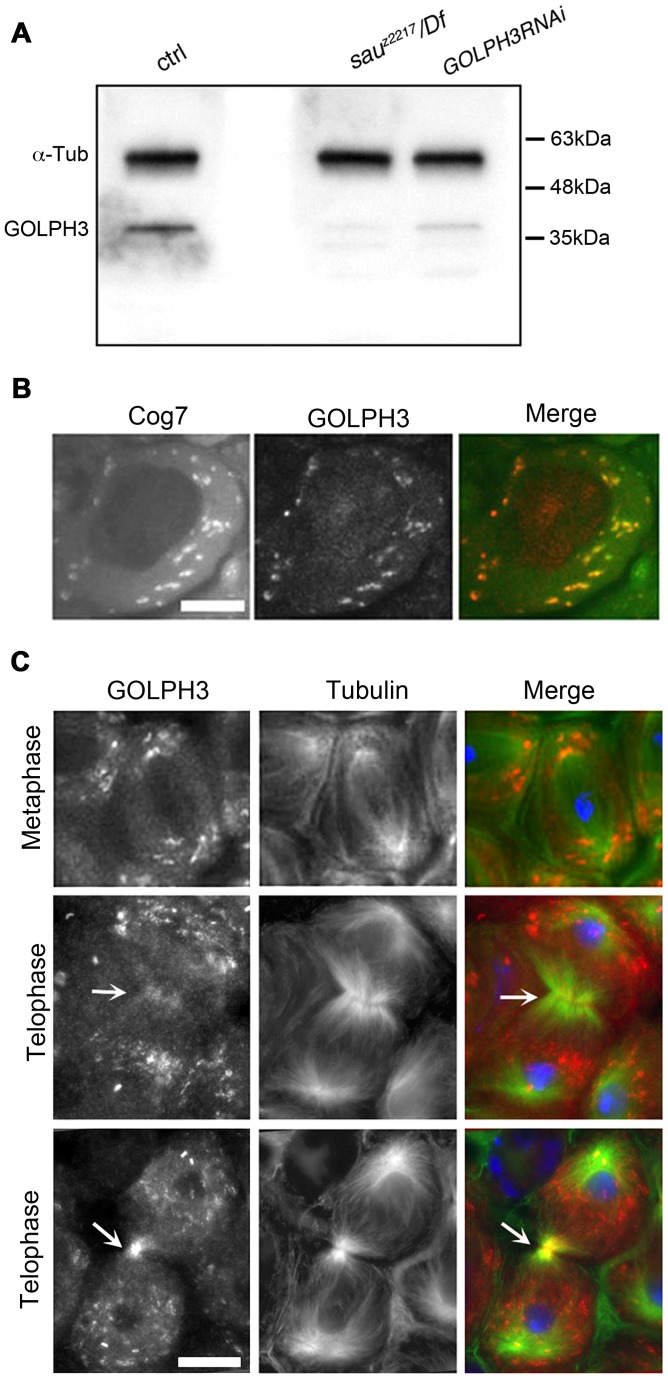
*Drosophila* GOLPH3 localizes to the cleavage furrow in dividing spermatocytes. (A) Western blotting from adult testis extracts. Polyclonal antibodies raised against GOLPH3 protein, recognized a band of the predicted molecular weight. The band is reduced in testis extracts from *sau* ***^z2217^***/*Df(2L)Exel7010* and *GOLPH3RNAi* males. α-Tubulin was used as a loading control. (B) Colocalization of GOLPH3 (anti-GOLPH3, red) with the Golgi protein Cog7-GFP (green) in interphase spermatocytes. (C) In dividing spermatocytes GOLPH3 protein (red) was enriched in Golgi derived vesicles and accumulated at the cleavage furrow (arrows) during telophase (green) Tubulin; (blue) DNA. Scale Bar; 10 µm.

Polyclonal antibodies raised against the entire *Drosophila* GOLPH3 protein, recognized a band of the predicted molecular weight in Western blots from extracts of adult testes (Figure 2A). This band appeared reduced in testis extracts from *GOLPH3RNAi* and *sau* ***^z2217^***/*Df* males indicating that the antibodies specifically reacted with *Drosophila* GOLPH3 (Figure 2A).

Immunofluorescence analysis reveals that the GOLPH3 protein localized to Golgi structures in spermatocytes. In interphase spermatocytes at stage S5 (see Ref. [29] for spermatocyte stages), GOLPH3 was enriched at multiple round structures that also contained the Golgi marker Cog7 [30] (Figure S1 and 2B). In dividing spermatocytes at metaphase and anaphase, GOLPH3 was associated with vesicles in the polar regions of the cell (Figure 2C). Strikingly, during telophase GOLPH3 protein accumulated at the cleavage furrow (Figure S1 and Figure 2C) consistent with a role in cytokinesis.

### Mutations in *Drosophila GOLPH3* affect the Golgi stack structure in interphase primary spermatocytes

**Figure 3:**
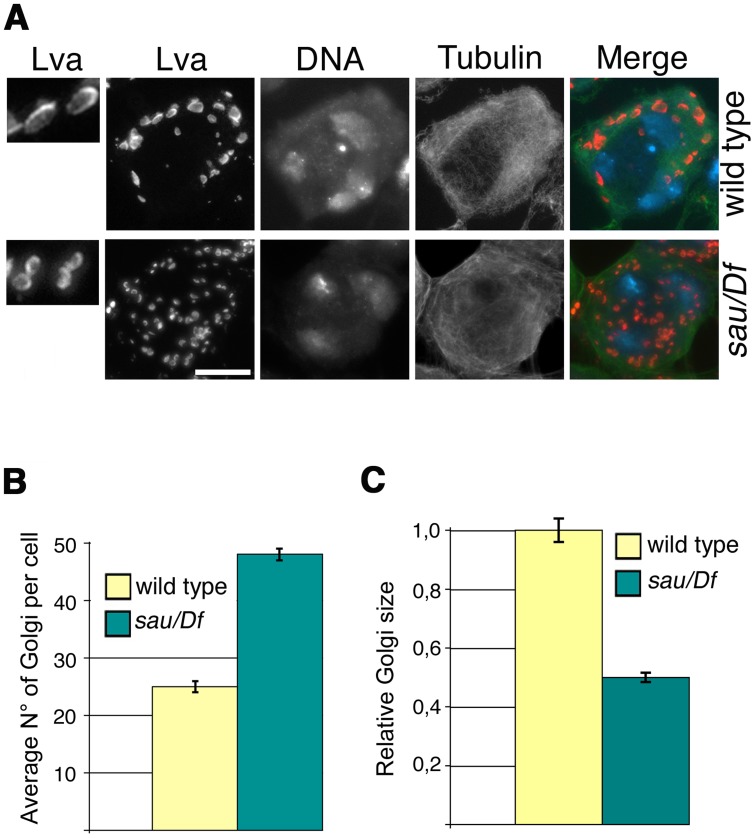
Mutations in *GOLPH3* affect Golgi structure in primary spermatocytes. (A) G2 spermatocytes during the S5 stage, stained for Lva (red), Tubulin (green) and DNA (blue). Spermatocytes at this stage are characterized by three distinct chromatin clumps (corresponding to each major bivalents) and an interphase microtubule cytoskeleton (absence of asters). Left panels show enlargements of Golgi stacks in Lva images. Note that in wild type the size of Lva fluorescent units is approximately two fold when compared to *sau^z2217^/Df(2L)Exel7010 (sau/Df)* mutants. Scale Bar; 10 µm (B) Average number of Golgi bodies per cell (± SEM), visualized by Lva staining in spermatocytes at S5 stage from wild type and *sau^z2217^/Df(2L)Exel7010 (sau/Df)* mutant males. (C) Average size (± SEM), of Golgi stacks quantified by image J (Area in 2D images), in spermatocytes immunostained for Lva at S5 stage. Measures were normalized to control average size (set at 1). See also Materials and Methods.

Consistent with the previous finding that depletion of human GOLPH3 affects Golgi architecture [16] GOLPH3 is essential for maintaining Golgi structure in *Drosophila* spermatocytes. In interphase wild type spermatocytes at stage S5, stained for the golgin Lava lamp (Lva, [31]), the average number of Golgi bodies per cell was 25 (Figure 3A and 3B). Conversely, spermatocytes from *sau* ***^z2217^***/*Df* mutant males, stained for Lva at the same stage, exhibited a 1.9- fold increase in the number of Golgi bodies (Figure 3A and 3B), with the average size decreased by 50% (Figure 3A and 3C). Previous work demonstrated that each Golgi in *Drosophila* has a paired structure consisting of two stacks held together through an actin-based mechanism [32]. The near-doubling of Golgi units observed in *sau* ***^z2217^***/*Df* mutant spermatocytes raises the possibility that GOLPH3 may play a role in maintaining the paired Golgi organization in interphase spermatocytes.

### GOLPH3 is required for central spindle and contractile ring formation

**Figure 4:**
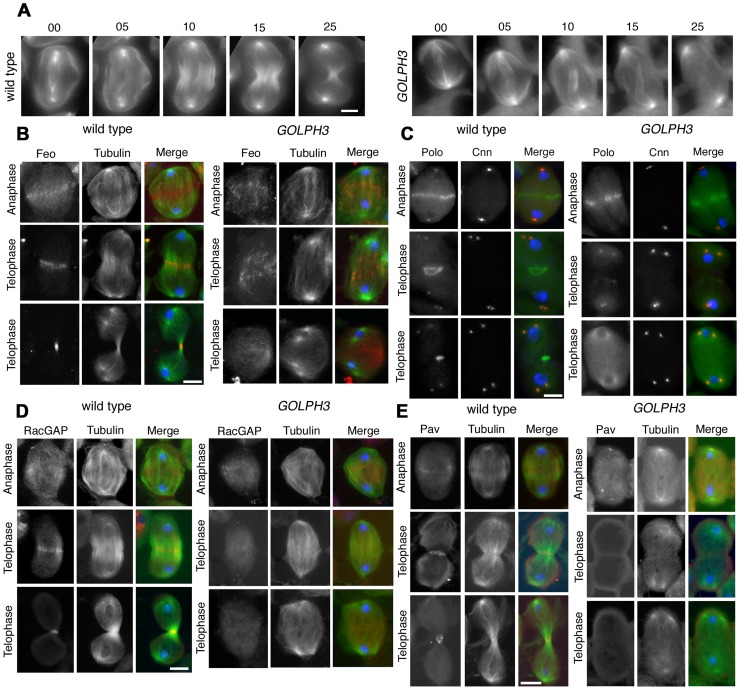
GOLPH3 is required for central spindle assembly and localization of the centralspindlin components at the cell midzone. (A) Time lapse analysis of central spindle formation in spermatocytes from wild type and *sau^z2217^/Df(2L)Exel7010 (GOLPH3*) expressing EGFP-β-tubulin. In wild-type dividing spermatocytes at early anaphase, peripheral microtubules (MT) probed the equatorial cortex and started bundling (Frames 0, 5). Bundling of this population of MTs was accompanied by cell elongation and bundling of interior MTs (Frame 10). Furrow ingression compacted the MTs arrays (Frames 15) giving rise to the characteristic telophase central spindle that was pinched in the middle during cytokinesis (Frame 25). In *sau^z2217^/Df(2L)Exel7010 (GOLPH3*), peripheral MTs contacted the cortex at cell equator during early anaphase (Frames 0, 5) but failed to stably interdigitate (Frames 10, 15). In addition interior MTs failed to bundle at the cell midzone (Frame 15) and cells failed to constrict (Frames 15, 25). (B) Anaphase and telophase spermatocytes from wild type and *sau^z2217^/Df(2L)Exel7010 (GOLPH3*) stained for Feo (red), Tubulin (green), and DNA (blue). (C) Anaphase and telophase spermatocytes from wild type and *sau^z2217^/Df(2L)Exel7010 (GOLPH3*) stained for Polo (green), Centrosomin (Cnn, red) and DNA (blue). (D,E) Anaphase and telophase spermatocytes from wild type and *sau^z2217^/Df(2L)Exel7010 (GOLPH3*) stained for Tubulin (green), DNA (blue) and either RacGAP50C (D) or Pavarotti (Pav) (E) (red). Scale Bar; 10 µm.

**Figure 5:**
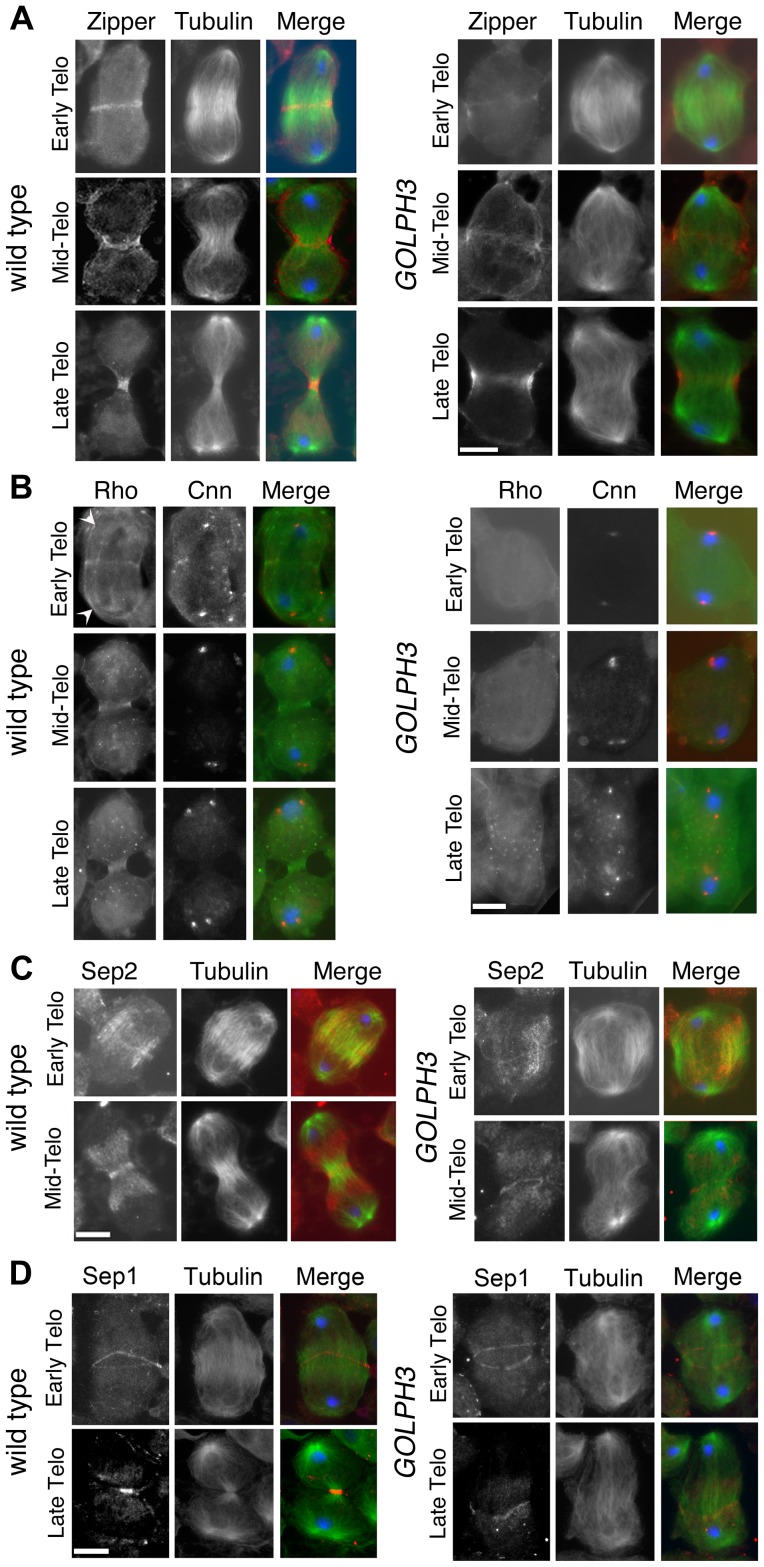
GOLPH3 is required for maintaining Rho1 at the equatorial cortex and stabilizing Septin and Myosin rings. (A) Wild type and *sau^z2217^/Df(2L)Exel7010 (GOLPH3*) spermatocytes during late anaphase-early telophase and mid-late telophase stained for Tubulin (green), DNA (blue) and the Myo II heavy chain Zipper (red). (B) Wild type and *sau^z2217^/Df(2L)Exel7010 (GOLPH3*) spermatocytes during late anaphase-early telophase and mid-late telophase stained for Centrosomin (Cnn, red), DNA (blue) and Rho1 (green). Arrowheads indicate enrichment of Rho1 at the astral membranes. (C,D) Wild type and *sau^z2217^/Df(2L)Exel7010 (GOLPH3*) spermatocytes during telophase stained for Tubulin (green), DNA (blue) and either Sep2 (C) or Sep 1 (D) (red). Scale Bar; 10 µm.

Previous analysis of *sau* ***^z2217^/*** *sau* ***^z2217^*** mutants, revealed defective central spindle in telophase spermatocytes [28]. Tubulin staining indicated defects in central spindle organization in *sau* ***^z2217^*** */Df* hemizygous mutant males (Figure 4 and 5). Analysis of the dynamics of central spindle formation, performed in wild type (n = 10) and *sau* ***^z2217^*** */Df* (n = 10) living spermatocytes expressing EGFP-β-tubulin [33], suggested a role for GOLPH3 in the initial steps of central spindle formation (Figure 4A). Localization of proteins associated with central spindle microtubules was also defective in *sau* ***^z2217^*** */Df* mutant spermatocytes undergoing meiotic division. In both wild type and *sau* ***^z2217^*** */Df* spermatocytes the microtubule bundling protein Fascetto/Prc1 (Feo, [34]) started to concentrate to microtubules at cell equator during anaphase (n = 44 mutant cells; n =  32 control cells; Figure 4B). However, in contrast to wild type, telophases from *sau* ***^z2217^*** */Df* mutants did not exhibit a tight equatorial Feo band; instead Feo staining was either in puncta scattered between the two nuclei, or appeared diffuse (n = 50 mutant cells; n = 45 control cells; Figure 4B). Similar results were obtained in spermatocytes stained for the Polo kinase (Polo, [35]). Although Polo concentrated at the midzone of *sau* ***^z2217^*** */Df* mutant spermatocytes during anaphase (n = 24 mutant cells; n = 24 control cells), it was dispersed in patches or diffuse during telophase (n = 34 mutant cells; n = 32 control cells; Figure 4C). Localization of the centralspindlin complex, which consists of the Rho GTPase activating protein (GAP) RacGAP50C [36] and the kinesin-like Pavarotti (Pav) [37], was also affected by loss of function of *GOLPH3*. *sau* ***^z2217^*** */Df* mutant spermatocytes displayed faint concentration of both RacGAP50C (n = 32 mutant cells; n = 34 control cells) and Pav (n = 38 mutant cells; n = 40 control cells) at peripheral microtubules during anaphase (Figure 4D and 4E). In mutant spermatocytes at mid-late telophase these proteins failed to accumulate at both peripheral and interior microtubules (n = 50 mutant cells and n = 46 control cells examined for RacGAP50C; n = 44 mutant cells and n = 50 control cells examined for Pav; Figure 4D and 4E).

Previous analysis failed to detect F-actin rings in most telophase spermatocytes from *sau* ***^z2217^*** */sau* ***^z2217^*** homozygous males [28]. Staining for F-actin using an improved protocol (see Materials and Methods) revealed defects in F-actin ring assembly in *sau* ***^z2217^*** */Df* mutant spermatocytes, with most telophases devoid of an F-actin ring (85%; n = 34 mutant cells; n = 44 control cells; Figure S2). *sau* ***^z2217^*** mutation also impaired formation of regular Myosin II (Myo II) cortical rings. The Myo II heavy chain Zipper [38] formed a cortical ring at cell equator of wild type cells at late anaphase-early telophase (100% of cells, n = 48; Figure 5A). Zipper rings appeared tighter and constricted in mid- late telophase (100% of cells, n = 78; Figure 5A). Most *sau* ***^z2217^*** */Df* mutant spermatocytes, fixed at late anaphase, displayed Zipper at cell equator. However in these cells Zipper was associated with thin rings at the equatorial cortex (n = 64; Figure 5A). In *sau* ***^z2217^*** */Df* spermatocytes fixed at mid-late telophase, Zipper was associated with patches at cell equator (60% of cells) or formed unconstricted fragmented rings (30% of cells) (n = 120; Figure 5A). Contractile ring dynamics was examined *in vivo* in dividing spermatocytes expressing the GFP-tagged-regulatory light chain Spaghetti Squash-GFP (Sqh-GFP, [38], Figure S3). In wild type anaphase spermatocytes, Sqh-GFP concentrated into an equatorial ring that underwent constriction immediately after assembly (n = 10). In all mutant spermatocytes examined (n = 8), Sqh-GFP failed to concentrate into a well formed ring (Figure S3) and cells failed to constrict.

Consistent with the defects in F-actin ring assembly at the plasma membrane, cortical accumulation of Rho GTPase at an equatorial position was defective in mutant dividing spermatocytes. In wild type spermatocytes at late anaphase-early telophase, Rho1 was enriched at the astral membranes and the parafusorial membranes and started to concentrate into a narrow ring at the equatorial cortex (n = 32; Figure 5B). The Rho1 ring appeared thicker in mid-telophase spermatocytes and fully constricted in late telophase (n = 36; Figure 5B). In contrast, in most dividing spermatocytes from *sau* ***^z2217^*** */Df*, Rho1 was diffuse in the cytoplasm and failed to concentrate into clear cortical rings at the cell equator during either late anaphase/early telophase (93%, n = 27; Figure 5B) or mid-late telophase (93%, n = 30; Figure 5B). Localization of Septins was also affected in *GOLPH3* mutants. These cytoskeletal scaffolding proteins, bind to phosphoinositides and form a membrane-associated filament system required to anchor the actomyosin ring to the plasma membrane [39]. In wild type dividing spermatocytes both Septin1 (Sep1, [40]; n = 25) and Septin2 (Sep2, [41]; n = 45) concentrated into thin equatorial rings during early telophase (Figure 5C and 5D). At later telophase stages, rings of both Sep2 (n = 63) and Sep1 (n = 44) appeared thicker as constriction progressed cells (Figure 5C and 5D). In *sau* ***^z2217^*** */Df* telophases Septins concentrated at the cell midzone (n = 42 cells examined for Sep2; n = 24 cells examined for Sep1; Figure 5C and 5D). However in mutant spermatocytes fixed at mid-late telophase Septin rings appeared thin and fragmented (n = 62 for Sep 2, n = 33 for Sep1; Figure 5C and 5D). Thus wild type function of GOLPH3 protein is required to maintain active Rho to the equatorial cortex and stabilization of Myo II and Septin-containing rings.

### PI(4)P binding is essential for GOLPH3 function in cytokinesis

**Figure 6:**
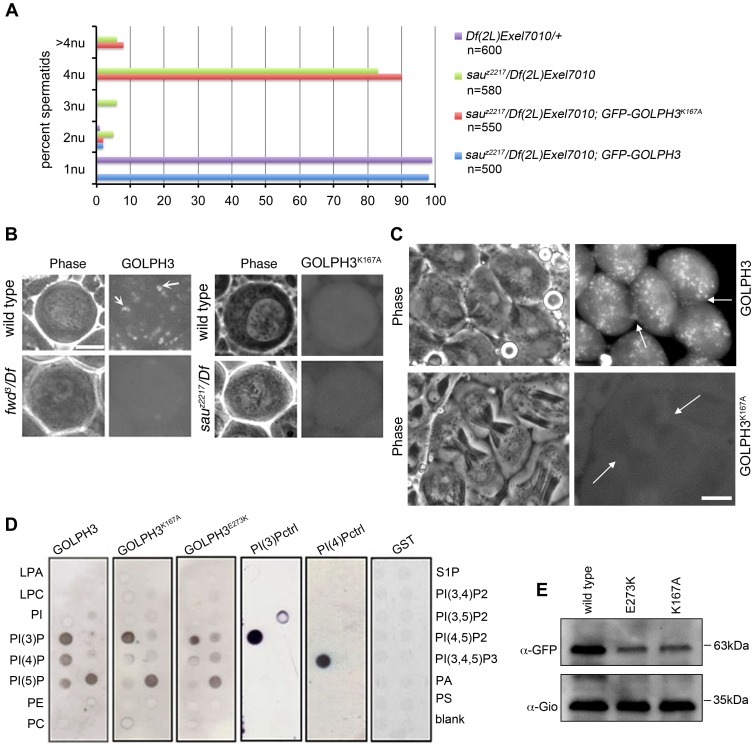
Binding to PI(4)P is essential for GOLPH3 function during cytokinesis. (A) Frequencies of spermatids (percentage) containing 1, 2, 3, 4 or more than 4 nuclei (nu) per Nk in testes from males of the indicated genotypes. (n); total number of spermatids counted per each genotype. Scale Bar; 10 µm. (B) Live spermatocytes of the indicated genotypes, expressing either GFP-GOLPH3 or a mutant version of GFP-GOLPH3 carrying K167A/R170L substitutions (GFP-GOLPH3^K167A^) were imaged at the same exposure time. GFP-GOLPH3 failed to localize to Golgi stacks (fluorescent round bodies indicated by arrows) in *fwd^3^/Df(3L)7C [fwd^3^/Df]* mutant spermatocytes. GFP-GOLPH3^K167A^ failed to localize at the Golgi membranes in both wild type and *sau^z2217^/Df(2L)Exel7010* (*sau^z2217^/Df*) mutant spermatocytes. (C) Phase-contrast and corresponding fluorescence images of live telophase spermatocytes expressing either GFP-GOLPH3 (GOLPH3) or GFP-GOLPH3 carrying K167A/R170L substitutions (GOLPH3K^167A^). Arrows indicate cleavage furrows. Scale Bar; 10 µm. (D) PIP strips (Echelon) were incubated with the indicated GST-tagged protein or GST, followed by anti-GST antibody for detection of lipid binding. PI(3)P ctrl and PI(4)P ctrl indicate GST-tagged control proteins that bind to either PI(3)P or PI(4)P. (E) Immunoblotting analysis of wild type testes expressing either GFP-GOLPH3 (wild type), GFP-GOLPH3^E273K^ (E273K) or GFP-GOLPH3^K167A/R170L^ (K167A). Giotto (Gio) was used as a loading control.

To test whether binding to PI(4)P is essential for GOLPH3 function in cytokinesis, we constructed a mutant version of GFP-GOLPH3 (GFP-GOLPH3^K167A/R170L^) carrying two substitutions (red arrows in Figure 1C) that, on the basis of crystal structure of human GOLPH3 [16], [17] are predicted to impair GOLPH3 binding to PI(4)P. GFP-GOLPH3^K167A/R170L^ was unable to rescue the cytokinetic phenotype of *sau* ***^z2217^*** (Figure 6A) and failed to localize to Golgi membranes and to the cleavage site in both wild type and *sau* ***^z2217^*** */Df* (Figure 6B and 6C). Localization of wild type GOLPH3 protein at Golgi stacks was abolished in spermatocytes from mutants in *fwd* (Figure 6B), further supporting the requirement for PI(4)P for GOLPH3 recruitment to this organelle. The Glu-Lys (E273K) mutation in our original *sau* ***^z2217^*** allele also affected the ability of GOLPH3 to bind to phosphoinositides. *Drosophila* wild type GOLPH3, and GOLPH3^K167A/R170L^, GOLPH3^E273K^ mutant proteins were expressed as GST-tagged proteins and their ability to bind phosphoinositides was assessed through protein-lipid overlay assays (Figure 6D). Wild type GOLPH3 protein interacted not only with PI(4)P but also with PI(3)P, PI(5)P and phosphatidic acid (PA, a glycerophospholipid) (Figure 6D). Remarkably, the pattern of lipid interaction for GOLPH3^E273K^ was comparable to that of GOLPH3^K167A/R170L^ indicating defective binding to both PI(4)P and PI(5)P (Figure 6D). Consistent with the defective PI(4)P lipid interaction of GOLPH3^E273K^ protein, GOLPH3 protein was not detected at the Golgi stacks of *sau^z2217^* mutant spermatocytes immunostained with anti-GOLPH3 antibodies (Figure S1). Moreover GOLPH3 failed to concentrate at the equatorial cortex of *sau^z2217^* dividing spermatocytes (Figure S1). To assess whether E273K and K167A/R170L mutations affect the stability of GOLPH3 protein the expression level of each GFP-tagged GOLPH3 protein was assessed by Western blot analysis. As shown in Figure 6E, in testes GFP-GOLPH3^E273K^ protein was decreased by 52% and GFP-GOLPH3^K167A/R170L^ protein by 54% relative to wild type GFP-GOLPH3. Together our data indicate that E273K and K167A/R170L mutations impair binding to PI(4)P and disrupt both the subcellular localization and the stability of GOLPH3 protein.

**Figure 7:**
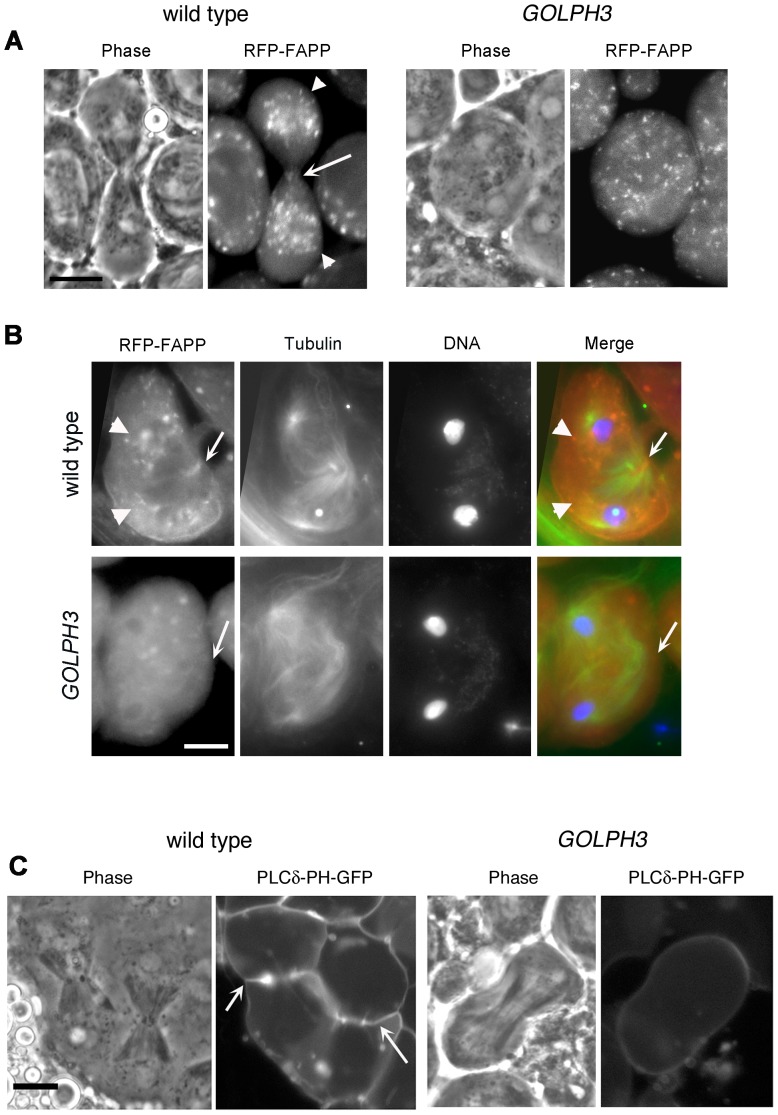
Localization of PI(4)P is disrupted in telophase cells from *GOLPH3* mutants. (A,C) Live spermatocytes expressing either RFP-PH-FAPP (RFP-FAPP) or PLCδ-PH-GFP were imaged at the same exposure time during telophase. Note that the distribution of both RFP-PH-FAPP and PLCδ-PH-GFP is affected in *GOLPH3* mutant spermatocytes indicating a defective PI(4)P and PI(4,5)P2 subcellular localization and/or synthesis. Arrows indicate accumulation of RFP-PH-FAPP (A) and PLCδ-PH-GFP (C) at the cleavage furrows of wild type cells. (B) Fixed telophase spermatocyes expressing RFP-PH-FAPP (RFP-FAPP) stained for RFP (red), tubulin (green) and DNA (blue). Arrows indicate the cell midzone. Arrowheads indicate the enrichment of RFP signals at the poles of the wild type cell. Scale Bar; 10 µm.

Reciprocally the localization of PI(4)P was disrupted in telophase cells of *sau* ***^z2217^*** */Df* spermatocytes. An RFP fusion to the PI(4)P marker PH-FAPP localized on Golgi organelles in both premeiotic and dividing spermatocytes (Figure 7A and data not shown) and was enriched at the midzone during telophase (Figure 7A and 7B) consistent with data of Polevoy and coauthors [15]. In premeiotic spermatocytes from *sau* ***^z2217^*** */Df*, RFP-PH-FAPP signals at the Golgi membranes, were as intense as in wild type (data not shown). However in dividing spermatocytes RFP-PH-FAPP signals were delocalized and failed to accumulate at the midzone during telophase (Figure 7A and 7B). Visualization of PI(4,5)P2, the phosphorylated derivative of PI(4)P, using PLCδ-PH-GFP [42] showed this lipid at the cleavage furrow plasma membrane in wild type but not in *GOLPH3* mutants (Figure 7C). Consistent with the idea that Fwd and GOLPH3 proteins contribute to the same process, double mutants carrying both *fwd* and *GOLPH3* mutations were synthetic lethal. Animals that were heterozygous for *GOLPH3* and hemizygous for *fwd* [*sau* ***^z2217^*** */+; fwd^3^/Df(3L)7C*] and animals that were heterozygous for *fwd* and homozygous/hemizygous for *GOLPH3* [*sau* ***^z2217^*** */sau* ***^z2217^*** *; fwd^3^/+* or *sau* ***^z2217^*** */Df(2L)Exel7010; fwd^3^/+*] were both viable. However, *sau* ***^z2217^*** was fully lethal in combination with *fwd^3^/Df(3L)7C*; individuals of genotypes *sau* ***^z2217^*** */Df(2L)Exel7010; fwd^3^/Df(3L)7C* or *sau* ***^z2217^*** */sau* ***^z2217^*** *; fwd^3^/Df(3L)7C* died during larval stages.

**Figure 8:**
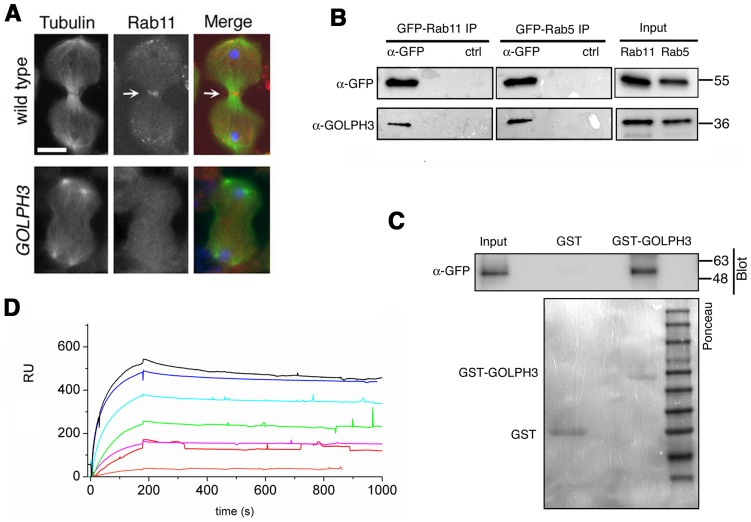
Rab11 forms a complex with GOLPH3 and depends on GOLPH3 for localization at the cleavage site. (A) Telophase spermatocytes from wild type and *sau^z2217^/Df(2L)Exel7010 (GOLPH3)* stained for Tubulin (green), Rab11 (red) and DNA (blue). Arrows indicate accumulation of Rab11 at the cleavage furrow of wild type spermatocyte. Scale Bar; 10 µm. (B) GOLPH3 protein coprecipitated with Rab11 and Rab5 in testis extracts. Protein extracts from testes expressing either GFP-Rab5 or GFP-Rab11 were immunoprecipitated with GFP-trap beads (α-GFP) and blotted for either GFP or GOLPH3. Control binding beads (ctrl) were used in control experiments. Input is 4% of lysates. Molecular masses are indicated in kilodaltons. (C) Bacterially expressed GST-GOLPH3 was purified by gluthatione-sepharose beads and incubated with testis lysates expressing GFP-Rab11. GST bound to gluthatione-sepharose beads was used as a negative control. GST-GOLPH3 precipitated GFP-Rab11 from testis protein extracts. Ponceau staining (Ponceau) is shown as loading control. Input is 4% of lysates. Molecular masses are indicated in kilodaltons. (D) Interaction of GOLPH3 immobilized on a CM5 sensorchip with Rab11, measured via Surface Plasmon Resonance experiments. Sensorgrams were obtained using GOLPH3 as ligand and Rab11 as analyte. Rab11 concentrations were as follows: 50 nM (orange), 200 nM (red), 400 nM (magenta), 1.0 µM (green), 2.0 µM (cyan), 4.0 µM (blue), 10.0 µM (black). The interaction of immobilized GOLPH3 ligand with the analyte was detected through mass concentration-dependent changes in the refractive index on the sensor chip surface, expressed as resonance units (RU). The increase in RU relative to baseline (0–180 s) indicates complex formation, whereas the decrease in RU represents dissociation of Rab11 from immobilized GOLPH3 upon injection of buffer. K_D_ = 180 nM indicates that a high affinity complex is formed; the dissociation kinetics are very slow (k_d_ about 1×10^−4^ s^−1^).

Like the requirement for Fwd for localization of both PI(4)P-associated secretory organelles and Rab11 at the cleavage furrow [15], [43], loss of *GOLPH3* also affected concentration of Rab11 at cell equator. In contrast to wild type, Rab11 was dispersed during cytokinesis in *sau* ***^z2217^*** */Df* spermatocytes (Figure 8A). GOLPH3 protein function was also required to localize other membrane trafficking markers at the cleavage furrow. In both wild type and *sau* ***^z2217^*** */Df* interphase spermatocytes at stage S5, a GFP fusion to clathrin light chain (Clc-GFP, [44]) was enriched in clusters of vesicular structures (Figure S4A). However, although Clc-GFP accumulated to cell equator of wild type telophase spermatocytes (Figure S4A) in *sau* ***^z2217^*** */Df* fluorescent signals were scattered and failed to accumulate at the cleavage furrow in cytokinesis (Figure S4A). Likewise analysis of a GFP-fusion to Rab5 [45], indicated that this protein, similarly to Rab11 and Clc-GFP, accumulated to the cleavage furrow in wild type but not in *sau* ***^z2217^*** */Df* (Figure S4B). The small GTPase proteins Rab5 controls early endocytic events such as clathrin-coated-vesicle-mediated transport from the plasma membrane to the early endosomes and homotypic early endosome fusion [46].

**Figure 9:**
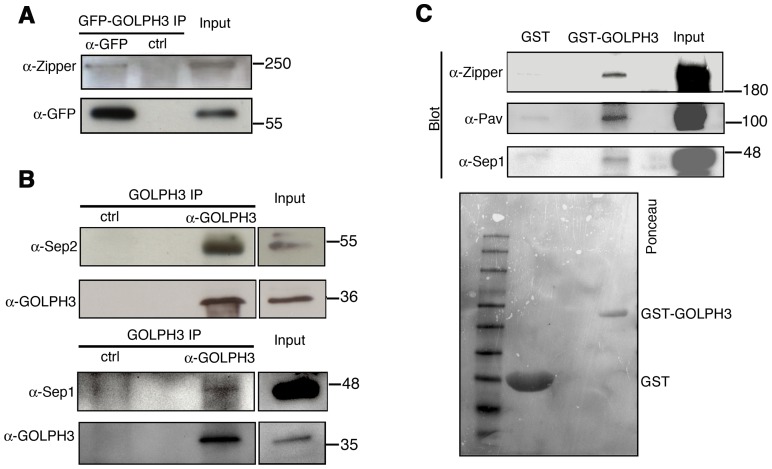
GOLPH3 protein interacts with both cytokinetic proteins in testis extracts. (A,B) Co-IP of GOLPH3 with Zipper and Septins. (A) Equal fractions of protein extracts from testes expressing GFP-GOLPH3 were immunoprecipitated with either mouse anti-GFP (α-GFP) or non-specific mouse IgG (ctrl) and blotted for either Zipper or GFP. (B) Protein extracts from wild type testes were immunoprecipitated with antibodies against *Drosophila* GOLPH3 (α-GOLPH3) and blotted for either Sep2, Sep1 or GOLPH3. Preimmune serum was used as control (ctrl). (C) Bacterially expressed GST-GOLPH3 was purified by gluthatione-sepharose beads and incubated with testis lysate. GST bound to gluthatione-sepharose beads was used as a negative control. GST-GOLPH3 precipitated Zipper, Pav and Sep1 from testis protein extracts. Ponceau staining (Ponceau) is shown as a loading control. In all the experiments input is 4% of lysates. Molecular masses are indicated in kilodaltons.

We next tested whether GOLPH3 interacts with central spindle and contractile ring components. Co-immunoprecipitation (Co-IP) and GST-pulldown experiments with testis extracts revealed that GOLPH3 interacts with the cytokinesis proteins Pav, Zipper and Septins (Figure 9A–9C and Figure S5B). We also explored whether GOLPH3 interacts with membrane trafficking proteins. Consistent with data from mammalian cells [18], we found that GOLPH3 and the retromer Vps35 protein form a complex also in *Drosophila* (Figure S5A and S5B). In addition we found that GOLPH3 protein interacts with Rab11 (Figure 8B and 8C) in *Drosophila* testes. Surface Plasmon Resonance (SPR) analysis indicated that Rab11 and GOLPH3 proteins form a high affinity, stable complex (Figure 8D), since the measured K_D_ was 180 nM, and the dissociation kinetics were very slow (k_d_ about 2×10^−4^ s^−1^). Finally Co-IP and GST-pulldown experiments indicated interactions of GOLPH3 protein with Rab5 and Clathrin in male germ cells (Figure 8B, S4C and S4D).

### *Drosophila* GOLPH3 is required for cytokinesis in larval brain neuroblasts

**Figure 10:**
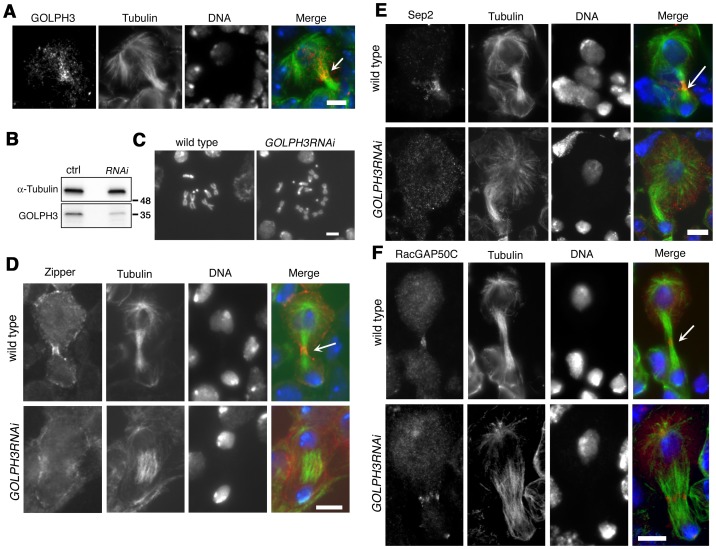
GOLPH3 is required for cytokinesis in larval neuroblasts. (A) GOLPH3 accumulates at the cleavage furrow of larval dividing neuroblasts. Larval neuroblast stained for Tubulin (green), GOLPH3 (red) and DNA (blue). Arrow indicates the midzone. Scale Bar, 5 µm. (B) Western blotting from larval brain extracts. Polyclonal antibodies raised against GOLPH3 protein, recognized a band of the predicted molecular weight. The band is reduced in larval brain extracts from animals expressing *UAS::GOLPH3RNAi* under control of *Tub-GAL 4 Tub-GAL80^ts^*. α-Tubulin was used as a loading control. Molecular masses are indicated in kilodaltons. (C) Normal male metaphase from wild type and tetraploid male metaphase from animals animals expressing *UAS::GOLPH3RNAi* under control of *Tub-GAL4 Tub-GAL80^ts^*. Scale Bar, 5 µm. (D,E) Knockdown of *GOLPH3* in larval neuroblasts affects assembly of a stable, fully constricted contractile ring and impairs formation of a compact central spindle. Localization of Zipper (D), Sep2 (E) and RacGAP50C (F) in late telophase neuroblasts from wild type animals and in animals expressing *UAS::GOLPH3RNAi* under control of *Tub-GAL 4 Tub-GAL80^ts^*, [*GOLPH3RNAi*]. Larval brains were stained for Tubulin (green), DNA (blue) and either Zipper, Sep 2 or RacGAP50C (red). Telophase stage is based on chromatin condensation. Arrows point to the midzone. Scale Bar, 5 µm.

The gene *GOLPH3* is essential for normal development and viability in *Drosophila*; *l(2)s5379* homozygotes or *l(2)s5379*/*Df(2L)Exel7010* hemizygotes die in early larval stages and individuals of genotype *sau* ***^Z2217^*** */Df* have a reduced life span when compared with control siblings. Moreover we found an early lethal phenotype in individuals carrying the *UAS::GOLPH3-RNAi* construct in combination with drivers for ubiquitous RNAi (such as Actin-GAL4 or tubulin-GAL4). We thus wondered whether GOLPH3 is required for cytokinesis also in somatic cells. In dividing neuroblasts of larval central nervous system (CNS) GOLPH3 was enriched at the cleavage furrow consistent with a requirement for cytokinesis (Figure 10A). To knockdown the gene *GOLPH3* in larval neuroblasts, *UAS::GOLPH3-RNAi* was expressed in larval CNS using the GAL4 system (tubulin-GAL4) with a temperature sensitive GAL80 (GAL80^ts^, [47]) (Figure 10B–10F). *GOLPH3* depletion in larval CNS resulted in a significant frequency of tetraploidy: 6% of metaphases were tetraploid in larval brains expressing dsRNA against *GOLPH3* (n = 1588 metaphases scored, Figure 10C) versus 0% in larval brains carrying the *UAS::GOLPH3-RNAi* transgene alone (n = 1800 metaphases scored). Immunostaining of larval brains for tubulin and Zipper also indicated cytokinesis defects in larval neuroblasts expressing *UAS::GOLPH3-RNAi* with 30% of mid-telophases failing to form a compact, fully constricted ring versus 0% in control cells (n = 150 mutant mid-telophases; n = 160 control mid-telophases; Figure 10D). Immunofluorescence analysis of mid-telophase neuroblasts depleted of *GOLPH3* also revealed failure to maintain Septin proteins at the cleavage furrow and to form a compact central spindle microtubule array (Figure 10E–10F).

## Discussion

In this paper we have provided the first compelling demonstration of the requirement for GOLPH3 protein function for cytokinesis. Our data demonstrate that the *Drosophila* homologue of human GOLPH3, plays an essential role in both contractile ring formation and vesicle trafficking. In most *GOLPH3* mutant dividing spermatocytes Rho1 appeared diffuse (this study) and F-actin rings failed to assemble (this study and [28]). *GOLPH3* mutation also prevented formation of robust Myosin II/Septin rings and disrupted central spindle assembly. GOLPH3 protein localized to the cleavage site during cytokinesis and biochemical analyses suggested interactions with Septins, Myosin and the centralspindlin component Pav.

Our observations of an enrichment of Sqh and Zipper at the cleavage site in the absence of a clear Rho1 ring are in agreement with a previous study on male germ cells depleted for anillin [48] and suggest that a pathway independent of Rho1 might be involved in the initial recruitment of Myosin II components in spermatocytes. Interestingly besides accumulating at the cleavage site, Rho1 is also enriched at the astral membranes in wild type dividing spermatocytes (arrowheads Figure 5B). It is then possible that a mechanism based on vesicle-mediated transport is implicated in maintaining Rho1 at the cleavage site during cytokinesis.

The requirement for GOLPH3 in spermatocyte cytokinesis is intimately connected to the ability of this protein to bind PI(4)P. A mutant version of GOLPH3 that was unable to associate with PI(4)P, failed to rescue the cytokinesis defects of *sau* ***^z2217^*** */Df* mutants. GOLPH3 depended on PI(4)P for its recruitment to both the Golgi and the cleavage furrow. Moreover PI(4)P concentration at the cleavage furrow required wild type function of GOLPH3.

Human GOLPH3 was shown to bind to PI(4)P and recruit MYO18A to the Golgi [16]. It has been proposed that PI(4)P, GOLPH3 and MYO18A are required for tethering Golgi membranes to the actin cytoskeleton to stretch the Golgi and produce a tensile force that facilitates vesicle budding [16]. We showed that GOLPH3 is required to maintain the integrity of paired Golgi stacks in *Drosophila* interphase spermatocytes. Remarkably a proteomic analysis of PI(4)P- containing liposomes demonstrated that these organelles are enriched in Rab11, PI 4-kinase IIIβ and the actin regulatory factors Rac1 and Scar/WAVE [49]. It is then likely that GOLPH3 participates in a PI(4)P dependent recruitment of these actin regulatory factors that contribute to regulate pairing of the Golgi stack structure. We speculate that a module containing PI(4)P-GOLPH3-MyoII is involved in producing a tensile force at the cleavage furrow required to shape the actomyosin ring architecture during cleavage furrow constriction.

**Figure 11:**
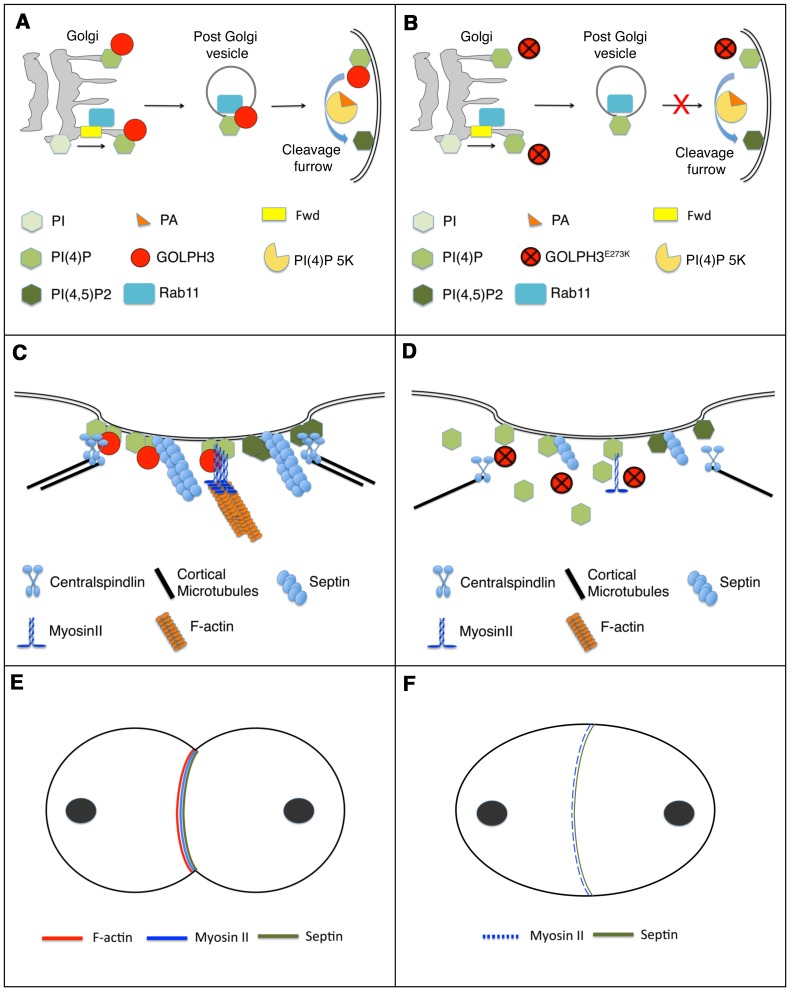
Diagram illustrating how GOLPH3 function might be involved in cytokinesis. (A,C,E), wild type; (B, D, F) *sau^z2217^/Df* mutants. (A) Golgi membranes are depicted in grey. Synthesis of PI(4)P at the Golgi requires function of the PI-4 kinase Fwd. Fwd also recruits Rab11 protein to Golgi where it becomes associated with secretory vesicles containing PI(4)P. GOLPH3 localization to the Golgi depends on the PI(4)P phosphoinositide. Wild type function of GOLPH3 is required for recruitment of PI(4)P-and Rab11-containing vesicles to the cleavage furrow. PI(4)P is the immediate precursor of PI(4,5)P2 that is generated in the cleavage furrow by PI(4)P 5-kinase. Binding to phosphatidic acid (PA), GOLPH3 might also contribute to PI(4)P 5-kinase activation and membrane curvature during cytokinesis. (B) In *sau^z2217^/Df* mutant cells GOLPH3 protein fails to concentrate to Golgi membranes and to the cleavage furrow. PI(4)P- and Rab11-containing vesicles fail to accumulate at the cleavage furrow. PI(4)P- 5 kinase [PI(4)P 5K] activation might be also impaired in *sau^z2217^/Df* mutants. (C) PI(4)P-GOLPH3 and PI(4,5)P2 regulate interaction of centralspindlin, septins and actomyosin with plasma membrane during cytokinesis. For the sake of simplicity, each cytokinesis protein is depicted separately. (D) In *sau^z2217^/Df* mutant cells PI(4)P-GOLPH3 does not localize to the cleavage site. As a result, localization of centralspindlin at the equatorial cortex is not maintained, centralspindlin-associated microtubules fail to stably bundle at the midzone and F-actin ring assembly is impaired. *sau^z2217^* mutations also affect stability of Myosin II and Septin filaments at the cleavage furrow. (E) In wild type telophase cells, rings containing F-actin, Myosin II and Septins constrict at the cleavage furrow during cytokinesis. (F) In *sau^z2217^/Df* mutant cells F-actin ring assembly fails, Myosin II and Septin rings are patchy or thin and fail to constrict.

A possible model to illustrate how GOLPH3 function might be involved in cytokinesis is depicted in Figure 11. Because GOLPH3 protein binds to both PI(4)P and Rab11 and both PI(4)P-and Rab11 were delocalized in *sau^z2217^* mutant spermatocytes, GOLPH3 protein might have a role in targeting PI(4)P- and Rab11-secretory vesicles to the cleavage furrow. PI(4)P is the substrate for PI(4)P 5-kinase for the synthesis of the PI(4,5)P2 lipid that is generated in the cleavage furrow and plays crucial roles during cytokinesis [10], [11]. Indeed imaging of dividing spermatocytes expressing PLCδ-PH-GFP, revealed enrichment of PI(4,5)P2 at the cleavage furrow in wild type but not in *GOLPH3* mutants. PI(4,5)P2 is known to enhance actin polimerization by modulating activity of proteins that regulate F-actin dynamics including profilin and cofilin [50], [51]. Thus, failure to assemble F-actin rings in *GOLPH3* mutants might be the consequence of a defective PI(4,5)P2 pool at the cleavage site. Recent data demonstrate that several cytokinesis players required in early stages including Rho, RhoGEF and centralspindlin subunit MgcRacGAP, also interact with phosphatidylinositol 4,5-bisphosphate [PI(4,5)P2] and/or PI(4)P and have plasma membrane interactions regulated by those phosphoinositides [52]–[55]. Defective PI(4,5)P2/PI(4)P concentration also affects Septin filament formation in the cleavage furrow. Septins form a gauze-like mesh tightly associated with plasma membranes [56]. Work in both mammalian and yeast cells demonstrated that Septins are able to interact *in vitro* with PIPs including P(4,5)P2, PI(3,4,5)P3 and PI(4)P through a polybasic domain [57]–[59]. Moreover Septin polymerization into filaments is enhanced by association with lipid bilayers [59], [60]. It has also been suggested that interaction of Septin filaments with phosphoinositides facilitates bending of plasma membranes and stabilizes the cleavage furrow [10]. In addition one of the mammalian septins (SEPT2) directly binds Nonmuscle Myo II and this interaction is required for Myo II full activation during cytokinesis [61].

The defects in cytokinesis in *GOLPH3* mutant spermatocytes also revealed malfunctioning of membrane trafficking pathways. Several studies have suggested an intimate connection between vesicle trafficking and actomyosin ring assembly/stability during cytokinesis. Syntaxin 1 is required for proper F-actin ring assembly in *Drosophila* S2 cells [62] and F-actin is targeted to the cleavage furrow of *Drosophila* postcellularized embryonic cells on endosomal vesicles [63]. In *Dyctiostelium* most clathrin null cells fail to undergo furrow ingression; in these cells MyoII is frequently associated with patches instead of forming a ring, a phenotype that is comparable to that of *GOLPH3* [64], [65]. Our data demonstrate that GOLPH3 protein is required for proper localization of Clathrin and Rab5 in dividing spermatocytes and might function as a molecular partner for both proteins in *Drosophila* male meiotic cells. A potential molecular interaction between Clathrin and GOLPH3 is further suggested by the presence in the GOLPH3 amino acid sequence of a putative clathrin binding motif [66], “LLDLD” indicated by a blue line in Figure 1C.

Because GOLPH3 not only binds to PI(4)P but also to PI(3)P and PI(5)P in lipid binding assays, we believe GOLPH3 protein may be also involved in membrane trafficking pathways that depend on phosphoinositides other than PI(4)P during cytokinesis. Indeed recent studies have involved PI(3)P in cytokinesis; PI(3)P positive endosomes localize to the midzone of both fungal and mammalian cells [67], [68] and inhibition of PI(3)P production results in cytokinesis failures in HeLa cells [69]. Strikingly, we have found that GOLPH3 protein also binds to the glycerophospholipid phosphatidic acid (PA) in lipid binding assays. It has been shown that PA is important for plasma membrane curvature [70] and is also an activator of PI(4)P 5-kinase [71]. Thus we speculate that may also contribute to PI(4)P 5-kinase activation and membrane remodelling during cytokinesis, binding to PA (Figure 11).

Our study demonstrates that GOLPH3 is essential for normal development and viability in *Drosophila*. In addition the presence of tetraploid metaphases and defective contractile rings in larval neuroblasts depleted of GOLPH3 indicates a requirement for this protein for cytokinesis of somatic cells. Because cytokinesis failures have been associated with premalignant disease and cancer [72], [73] our findings suggest novel insight into molecular circuits that involve the oncogene *GOLPH3* in cytokinesis.

## Materials and Methods

### Fly stocks and transgenes

The *sau^z2217^* allele was from C. Zuker collection of ethylmethane sulfonate-induced lines that were screened for defects in spermatocyte cytokinesis [28]. The chromosomal deficiencies *Df(2L)Exel6007*, *Df(2L)Exel7010, Df(3L)7C* and the P element *l(2)s5379* were obtained from the Bloomington Drosophila Stock Center (Indiana University, Bloomington, IN). Flies carrying the *fwd^3^* mutant allele and flies expressing GFP-Cog7 were previously described [12], [30]. *UAS::GOLPH3-RNAi* flies were from the Vienna Drosophila RNAi Collection (VDRC line 46150). *Bam-GAL4* [74] was used to inactivate *GOLPH3* in spermatocytes and to drive the expression of Chc-RFP from the *UAS::Chc-RFP* transgene. *P{tubP-GAL4}LL7 (tub-GAL4)* flies were from the Bloomington Drosophila Stock Center. Flies carrying both *tub-GAL4* and *tubGAL80^ts^* transgenes (on third chromosome) were kindly provided by Timothy Megraw (University of Florida).

The following GFP/RFP transgenic lines were a gift from H.C. Chang (Pardue University): *β2*t*::GFP-Rab5*, *β2*t::*GFP-Rab11*, *β2*t::*Clc-GFP*, *UAS::Chc-RFP* [44], [45]. The β-tubulin-EGFP stock [33] was kindly provided by D. Glover (University of Cambridge, UK). The Sqh-GFP stock [38] was a gift from R. Karess (University of Paris, Paris France). The RFP-PH-FAPP and PLCδ-PH-GFP strains [15], [42] were kindly provided from J. A. Brill (University of Toronto).

### Molecular cloning

To identify the mutation in the EMS-induced *sau^z2217^* allele, genomic DNA corrisponding to *CG7085/GOLPH3* gene was amplified by PCR and sequenced on both strands (Bio-Fab research service). DNA sequences from *sau^z2217^*/*sau^z2217^* individuals were compared to sequences of the original Zuker-background chromosome to confirm the missense mutation. To generate the *GFP-GOLPH3* fusion construct, the EGFP CDS was fused in frame to the aminoterminus of the full length cDNA corrisponding to *CG7085* gene and cloned into the pCaSpeR4 under the control of α-tubulin promoter (pCaSpeR4-tubulin, see Ref. [75] for details on this vector). To generate the Vps35-mRFP transgene, DNA encoding Vps35-mRFP was PCR amplified from a Vps35-mRFP expression plasmid (gift from Viktor Korolchuk, [76]) and cloned into pCaSper4-tubulin. To generate the Vps35-FLAG transgene, the cDNA of Vps35 was cloned into pCaSper4-tubulin in frame with C-terminal 3XFLAG sequence. Transgenic flies were generated by P-element mediated germline transformation, performed by Bestgene Inc. (Chino Hills, CA). *GFP-GOLPH3* was crossed into the *sau^z2217^* mutant background to test for phenotypic rescue of male sterility and meiotic cytokinesis failure. S2 cultures were transfected with Vps35-FLAG and either GFP or GFP-GOLPH3 plasmids using Cellfectin II Reagent (Invitrogen) as recommended by the manufacturer. Site-directed mutagenesis, used to generate K167A/R170L substitutions, was performed using the QuikChange Site-Directed Mutagenesis Kit (Stratagene). DNA constructs obtained after mutagenesis were confirmed by DNA sequencing on both strands. To obtain gluthatione S-transferase (GST) fusion proteins, full-length cDNAs corrisponding to either *Drosophila* wild type GOLPH3, GOLPH3^K167A/R170L^ or GOLPH3^E273K^, were cloned into a pGEX-6p-2 (GE Healthcare) at the GST C-terminus.

### Protein expression and purification, antibody generation

GST-full-length *Drosophila* GOLPH3 (dGOLPH3) proteins and GST-full-length *Drosophila* Rab11 were expressed in BL21-CodonPlus [DE3] cells (Invitrogen) and purified using HiTrap affinity columns (GTtrap FF, and GSTtrap HP columns, GE Healthcare) operated with AKTA 900 Fast Protein Liquid Chromatography. Polyclonal antisera were raised against the purified GST-dGOLPH3 protein. Immunization was carried out at Agro-Bio Services (www.agro-bio.com); two rabbits and two mice were injected using standard procedures. The anti-GST-GOLPH3 antisera were first depleted of anti-GST antibodies and then affinity-purified against GST-GOLPH3.

### Lipid binding assay

PIP Strips were purchased from Echelon (Echelon Biosciences) and were used following the manufacturer's instructions. Briefly, GST and GST fusion proteins, expressed and purified following the protocol described above, were incubated with PIP Strips at the concentration recommended by Echelon. Control GST-fusion proteins, for testing binding to a specific phosphoinositide, were purchased from Echelon and used at the recommended concentration. To detect the bound protein to PIP Strips, the following reagents were used: mouse anti-GST antibody, (1∶2000, Sigma #G-1160), anti mouse IgG conjugated with Horseradish peroxidase (HRP), (1∶5000, Sigma #A-9917); ECL Prime Western Blotting Detection Reagent (GE Healthcare).

### Western blotting and immunoprecipitation

For immunoblotting analysis of GOLPH3 protein, 40 adult testes from males of each genotype, were homogenized in 100 µl of Lysis buffer (10 mM Tris-HCl pH 7.5, 150 mM NaCl, 0.5 mM EDTA, 0.5% NP40, 1 mM PMSF, 1× Protease inhibitor Cocktail) using a Dounce homogenizer, for 40 minutes on ice. Cell lysates were cleared by centrifugation and protein concentration of supernatants was determined using the Bradford protein assay (Bio-Rad, Hercules CA). Equal amounts of protein extracts were analysed by SDS-PAGE and Western blotting. Samples were separated on Mini-protean TGX precast gels (Bio-Rad) and blotted to PVDF membranes (Bio-Rad). Membranes were blocked in Tris-buffered saline (Sigma-Aldrich) with 0,05% Tween-20 (TBST) containing 5% nonfat dry milk (Bio-Rad; Blotting GradeBlocker) for 3–4 hours at room temperature followed by incubation with primary and secondary antibodies diluited in TBST. For the coimmunoprecipitation (Co-IP) experiment shown in Figure 9A, 400 adult testes expressing GFP-GOLPH3 were homogenized in 500 µl of Lysis buffer (see above) for 40 minutes on ice. Lysate was clarified by centrifugation and protein concentration was quantified using the Bradford protein assay. 4% of lysate was retained as the “input”, the remainder was precleared with Sepharose-Protein G (Sigma) and divided into two. Fractions were incubated with either 2 µg of mouse monoclonal anti-GFP antibody (Roche) or 2 µg of non-specific mouse IgG (Sigma) for 2 hours at 4°C, followed by the addition of 40 µl of protein G-sepharose beads for further two hours. The beads were washed extensively with Lysis buffer, boiled in SDS sample buffer and separated by SDS-PAGE. To immunoprecipitate *Drosophila* GOLPH3 (dGOLPH3), testis lysate from 400 adult testes was precleared and divided into two. Fractions were incubated with either 5 µg of mouse anti-dGOLPH3 antibody S11047/1/56 or 5 µg of mouse pre immune serum (S11047/1), from the same animal before the immunization). After antibody incubation, immunoprecipitation was performed using the immunoprecipitation kit-Protein G (Roche) following the manufacturer's instructions. Other Co-IP experiments from testes expressing GFP and/or RFP-tagged proteins, were performed using the GFP/RFP trap-A kits and control binding beads purchased from ChromoTek (Planegg-Martinsried), following the protocol that was previously described [30]. Primary antibodies, used for immunoblotting were as follows: Rabbit anti-dGOLPH3, (1∶2500; G49139/77 this study), mouse monoclonal anti-α-Tubulin (1∶5000) (Sigma-Aldrich T6199), Rabbit anti-Zipper (1∶2000), gift from R.E. Karess; Rabbit anti-Pav (1∶2000), gift from D. Glover (University of Cambridge); Rabbit anti-Sep1 (1∶300) and Rabbit anti-Sep2 (1∶500) gift from J. Pringle (Stanford University, CA); Rabbit anti-Gio (1∶2000); Rat monoclonal anti-RFP (1∶1000), (ChromoTek, 5F8); mouse HRP anti-GFP (1∶1000), (Vector-Lab); mouse HRP anti-Flag (1∶1000), (clone M2 Sigma). HRP-conjugated secondary antibodies (GE Healthcare) were used at 1∶5000. After incubation with the antibodies, blots were washed in TBST and imaged using ECL detection kit (GE Healthcare).

### GST pulldown assays

GST and GST-GOLPH3 proteins were expressed in bacteria and purified using Glutathione-Sepharose 4B beads (GE Healthcare) following the manufacturer's instructions. To obtain testis protein extracts, at least 600 adult testes were homogenized for 40 minutes on ice in 500 µl of Lysis buffer (25 mM Tris-HCl pH 7.4, 150 mM NaCl, 0,5% NP-40, 1 mM EDTA) with the addition of Protease and phosphatase inhibitors cocktails (Roche), using a Dounce homogenizer. Procedures to obtain lysates from S2 cells, were previously described [75]. Lysates from either testes or S2 cells were cleared by centrifugation and protein concentration of the supernatants was determined by Bradford Assay (Bio-Rad). GST-pulldown was performed by incubating lysates with either GST or GST-GOLPH3 (at the appropriate concentration) bound to Glutathione-Sepharose 4B beads, at 4°C for two hours, with gentle rotation. The beads were washed three times in “wash buffer” (25 mM Tris-HCl pH 7.4, 150 mM NaCl, 1% NP-40, 1 mM EDTA, Protease and phosphatase inhibitors), boiled in SDS sample buffer, and separated by SDS-PAGE. The bound proteins were analysed by Western Blotting (see above). Before immunoblotting PVDF membranes were stained with Ponceau S (Sigma-Aldrich) as loading control.

### Surface Plasmon Resonance experiments

For Surface Plasmon Resonance (SPR) experiments, following elution of the GST fusion proteins from Glutathione Sepharose, the eluate was dialysed extensively against Cleavage Buffer (20 mM TRIS pH 7.0, 150 mM NaCl, 1 mM DTT). Digestion was performed with 2 units PreScission Protease for each 100 µg of fusion protein in the eluate, for 4 hours at 4°C. Once digestion was complete, the sample was applied to Glutathione Sepharose pre-equilibrated in Cleavage Buffer, to remove the GST portion of the fusion protein and the PreScission Protease from the protein of interest. Cleaved proteins were recovered from the flowthrough, and concentrated using 10 K Amicon Ultra-15 Centrifugal Filter Units. SPR experiments were carried out using a BIACORE X system (BIAcore AB, Uppsala, Sweden). The sensor chip (CM5, Biacore AB) was activated chemically by a 35 µl injection of a 1∶1 mixture of *N*-ethyl-*N*′-(3-(diethylaminopropyl) carbodiimide (200 mM) and *N*-hydroxysuccinimide (50 mM) at a flow rate of 5 µl/min. GOLPH3 was immobilized on the activated sensor chip via amine coupling. The immobilization was carried out in 20 mM sodium acetate at pH 5.2; the remaining ester groups were blocked by injecting 1 M ethanolamine hydrochloride (35 µl). This procedure ensures immobilization of GOLPH3 principally via the N-terminus. As a control, the sensor chip was treated as described above in the absence of GOLPH3. The level of immobilization of GOLPH3 was about 1200 RU. Rab11 (in 10 mM Hepes pH 7.4, 150 mM NaCl +0,005% surfactant P20) was injected on the sensor chip at a constant flow (30 µl/min). The interaction of immobilized GOLPH3 ligand with the analyte was detected through mass concentration-dependent changes in the refractive index on the sensor chip surface, expressed as resonance units (RU). The increase in RU relative to baseline indicates complex formation; the plateau region represents the steady-state phase of the interaction, whereas the decrease in RU represents dissociation of Rab11 from immobilized GOLPH3 after injection of buffer. A response change of 1000 RU typically corresponds to a change in the protein concentration on the sensor chip of 1 ng/mm^2^. The sensorgrams were analyzed by BIAevaluation software.

### Microscopy image acquisition and immunofluorescence

Cytological preparations were made with brains from third instar larvae and testes from either third instar larvae or adults. *sau^z2217^/Df(2L)Exel7010* mutants were used in all the immunofluorescence experiments for *GOLPH3* testes. To deplete GOLPH3 in larval neuroblasts *UAS::GOLPH3-RNAi* flies were crossed to *tub-GAL4 tubGAL80^ts^* flies. Progeny was kept at 18°C until the third instar stage and subsequently transferred to 29°C for 18 h before the analysis of the cytokinetic phenotype.

To visualize α Tubulin with either Clc-GFP or RFP-PH-FAPP, Lva with GFP-Cog7 or to stain F-actin with Rhodamine-phalloidin (Invitrogen), larval testes were fixed in 4% formaldehyde as previously described [30]. To visualize α Tubulin and GOLPH3, larval testes or brains were fixed and stained following the protocol described by Starr and coauthors [77]. Briefly testes and brains were dissected in 0.7% NaCl and transferred into a drop of PHEMT (60 mM Pipes, 25 mM HEPES, pH 7.0, 10 mM EGTA, 4 mM MgSO4, 0.5% Triton X-100) for two minutes. Testes and brains were then transferred to 4 µl of PHEMT containing 3.7% formaldehyde on a coverslip, gently squashed on an inverted slide and fixed for 10 minutes before immersing in liquid nitrogen. After removing the coverslip, preparations were immersed in methanol for 20 minutes (−20°C) and rehydrated in PBS containing 0.1% Triton for 20 minutes at room temperature. For immunostaining with other antibodies, preparations were fixed using 3.7% formaldehyde in PBS and then squashed in 60% acetic acid as previously described [43]. Monoclonal antibodies were used to stain α-Tubulin (1∶300; Sigma-Aldrich, T6199), RFP (1∶200; ChromoTek, 5F8); Rho1 (1∶100, p1D9 [78]; Developmental Studies Hybridoma Bank, University of Iowa), and Polo (1∶20; Mouse anti-Polo M294; [35]) gift from D. Glover. Polyclonal antibodies were as follows: Rabbit anti-dGOLPH3, (1∶2500; G49139/77 this study), rabbit anti-Zipper (1∶400; [38]), gift from R.E. Karess, rabbit anti-Lva (1∶500; [31]), gift from O. Papoulas (University of Texas); rabbit anti-Rab11 (1∶30; [79]), gift from M. Gonzalez-Gaitan (Max Planck Institute of Molecular cell Biology, Dresden) anti-Pav (1∶200; [37]) and anti-RacGAP50C (1∶200; [36]) kindly provided by D. Glover; anti-Feo (1∶200; [34]), gift from F. Vernì (Università Sapienza, Roma); anti-Sep1 and anti Sep2 (1∶30; [40], ). Alexa 555-conjugated anti rabbit IgG (Molecular Probes) and rhodamine/FITC-conjugated anti-mouse IgG (Jackson Immunoresearch), were used as secondary antibodies (1∶250 and 1∶20 respectively). Preparations fixed to visualize Clc-GFP together with tubulin, were first incubated with monoclonal anti-α–Tubulin (see above) followed by incubation with GFP-Booster (ChromoTek) diluited 1∶100 in PBS containing rhodamine-conjugated anti-mouse IgG. To visualize α-Tubulin together with F-actin, preparations were first incubated with anti α-Tubulin followed by incubation with Rhodamine-phalloidin (Invitrogen) diluited 20 U/ml in PBS containing FITC-conjugated anti-mouse IgG. In all cases preparations were mounted in Vectashield medium with DAPI (Vector labs). Images were captured with a charged-coupled device (CCD camera, Photometrics Coolsnap HQ), connected to a Zeiss Axioplan, epifluorescence microscope, equipped with an HBO 100-W mercury lamp, and 40× or 100× objectives as previously described [13], [43]. The number of Golgi stacks per cell was counted manually, by analyzing images of G2 spermatocytes at S5 stage, stained for tubulin, Lva and DNA. In total, 32 cells were examined for both wild type and *sau^z2217^/Df(2L)Exel7010*, in images captured in 4 duplicated experiments (n = 8 representative cells per each experiment). The size of Golgi stacks in spermatocytes was analysed using image J software (NIH; http://rsbweb.nih.gov/ij/). In total, 110 randomly selected Golgi were measured for both wild type and *sau^z2217^/Df(2L)Exel7010* using Image J by manual demarcation with a limiting polygon and calculation of its area (n = 4 duplicated experiments). Each measure was normalized to control average size (set at 1). To visualize mitotic chromosomes, larval brains were dissected in NaCl 0.7%, treated with hypotonic solution for 7 minutes and fixed in 45% acetic acid. Preparations were then immersed in liquid nitrogen, processed as per [28] and mounted in Vectashield medium with DAPI (Vector Laboratories, Burlingame, CA).

### Live imaging

Larval testes were prepared and imaged for time lapse as previously described [43]. Spermatocytes were examined with a Zeiss Axiovert 20 microscope equipped with a 63×, 1. 25 NA and a 63×, 1.4 NA objectives and a filter wheel combination (Chroma Technology Corp.). Images were collected at 1 minute time intervals with a CoolSnap HQ camera (Photometrics) controlled through a Metamorph software (Universal imaging); nine to eleven fluorescent optical sections were captured at 1-µm z steps and maximally projected using the Metamorph software.

## Supporting Information

Figure S1 GOLPH3 protein fails to localize to the Golgi and to the midzone of *sau^z2217^/Df(2L)Exel7010* mutant spermatocytes. Wild type and *sau^z2217^/Df(2L)Exel7010 (sau^z2217^/Df)* mutant spermatocytes during interphase (A) and telophase (B) stained for GOLPH3 (red), tubulin (green) and DNA (blue). White arrowheads in A point to Golgi stacks. White arrowhead in B indicates accumulation of GOLPH3 at the cleavage furrow of wild type telophase. Black arrowheads in B point to GOLPH3-enriched vesicles at the poles of wild type cells. Centriole staining (white arrows) by anti-GOLPH3 is not specific. Scale Bar, 10 µm.
(TIF)

Figure S2 Formation of F-actin rings fails in spermatocytes from *sau^z2217^/Df(2L)Exel7010* males. Wild type and *sau^z2217^/Df(2L)Exel7010 (sau^z2217^/Df)* mutant spermatocytes stained for F-actin (red), Tubulin (green) and DNA (blue) at telophase I. Arrow in wild type indicates the F-actin ring. Note the defective central spindle and the absence of a cortical F-actin ring (Arrow) in mutant cell. Scale Bar, 10 µm.
(TIF)

Figure S3 Dividing spermatocytes from *sau^z2217^/Df(2L)Exel7010* (*GOLPH3*) mutant males fail to form Sqh-GFP containing rings. Frames from time-lapse sequences of spermatocytes expressing Sqh-GFP undergoing cytokinesis. Time 0 corresponds to the earliest detection of Sqh-GFP at the cell equator. Scale Bar, 10 µm.
(TIF)

Figure S4 Mutations in *GOLPH3* impair recruitment of endocytic markers to the cleavage furrow. (A) *GOLPH3* mutations affected concentration of Clathrin at the cleavage furrow. Spermatocytes from wild type and *sau^z2217^/Df(2L)Exel7010 (GOLPH3)* males expressing Clc-GFP, fixed and stained for Tubulin (green) and DNA (blue), and GFP (GFP-Booster, red). Arrows indicate clusters of vesicular structures in interphase spermatocytes. Arrowhead indicates accumulation of Clc-GFP at the midzone of wild type telophase. Note that in mutant telophase, Clc-GFP-containing organelles appear tiny and scattered in the cytoplasm. Scale Bar, 10 µm. (B) Phase-contrast and corresponding fluorescence micrographs of telophase spermatocytes expressing GFP-Rab5. Arrow indicates accumulation of GFP-Rab5 at the cleavage furrow of wild type spermatocyte. Scale Bar, 10 µm. (C) GOLPH3 protein coprecipitated with Clathrin heavy chain (Chc) in testis extracts. Protein extracts from testes expressing Chc-RFP were immunoprecipitated with RFP-trap beads (α-RFP) and blotted for either RFP or GOLPH3. Control binding beads (ctrl) were used in control experiments. Input is 4% of lysates. Molecular masses are indicated in kilodaltons. (D) Bacterially expressed GST-GOLPH3 was purified by gluthatione-sepharose beads and incubated with testis lysates expressing Clathrin light chain tagged with GFP (Clc-GFP). GST bound to gluthatione-sepharose beads was used as a negative control. GST-GOLPH3 precipitated Clc-GFP from testis protein extracts. Ponceau staining (Ponceau) is shown as a loading control. Input is 4% of lysates. Molecular masses are indicated in kilodaltons.
(TIF)

Figure S5 Vps35 protein coprecipitates with GOLPH3 in *Drosophila* S2 cells. (A) S2 cells were transiently transfected with a construct expressing Vps35-Flag. Bacterially expressed GST-GOLPH3 was purified by gluthatione-sepharose beads and incubated with S2 cells expressing Vps35-Flag. GST bound to gluthatione-sepharose beads was used as a negative control. GST-GOLPH3 precipitated Vps35-Flag from S2 cell extracts. Ponceau staining (Ponceau) is shown as a loading control. (B) Zipper and Vps35-Flag coprecipitated with GFP-GOLPH3 in *Drosophila* S2 cells. S2 cells were transiently transfected with a construct expressing Vps35-Flag and with either a construct expressing GFP or a construct expressing GFP-GOLPH3. Extracts from S2 cells expressing Vps-Flag and either GFP or GFP-GOLPH3 were immunoprecipitated with anti-GFP (GFP-trap) and blotted for either Zipper (α-Zipper) or Vps35-Flag (α-Flag). Western blot at the top of this panel shows the level of expression of the GFP proteins (input).
(TIF)
