# Supplementary figures and images for: GOLPH3 Is Essential for Contractile Ring Formation and Rab11 Localization to the Cleavage Site during Cytokinesis in Drosophila melanogaster
Source: PLoS Genet. 2014 May 1;10(5):e1004305. doi: 10.1371/journal.pgen.1004305 (PMC4006750; doi:10.1371/journal.pgen.1004305)

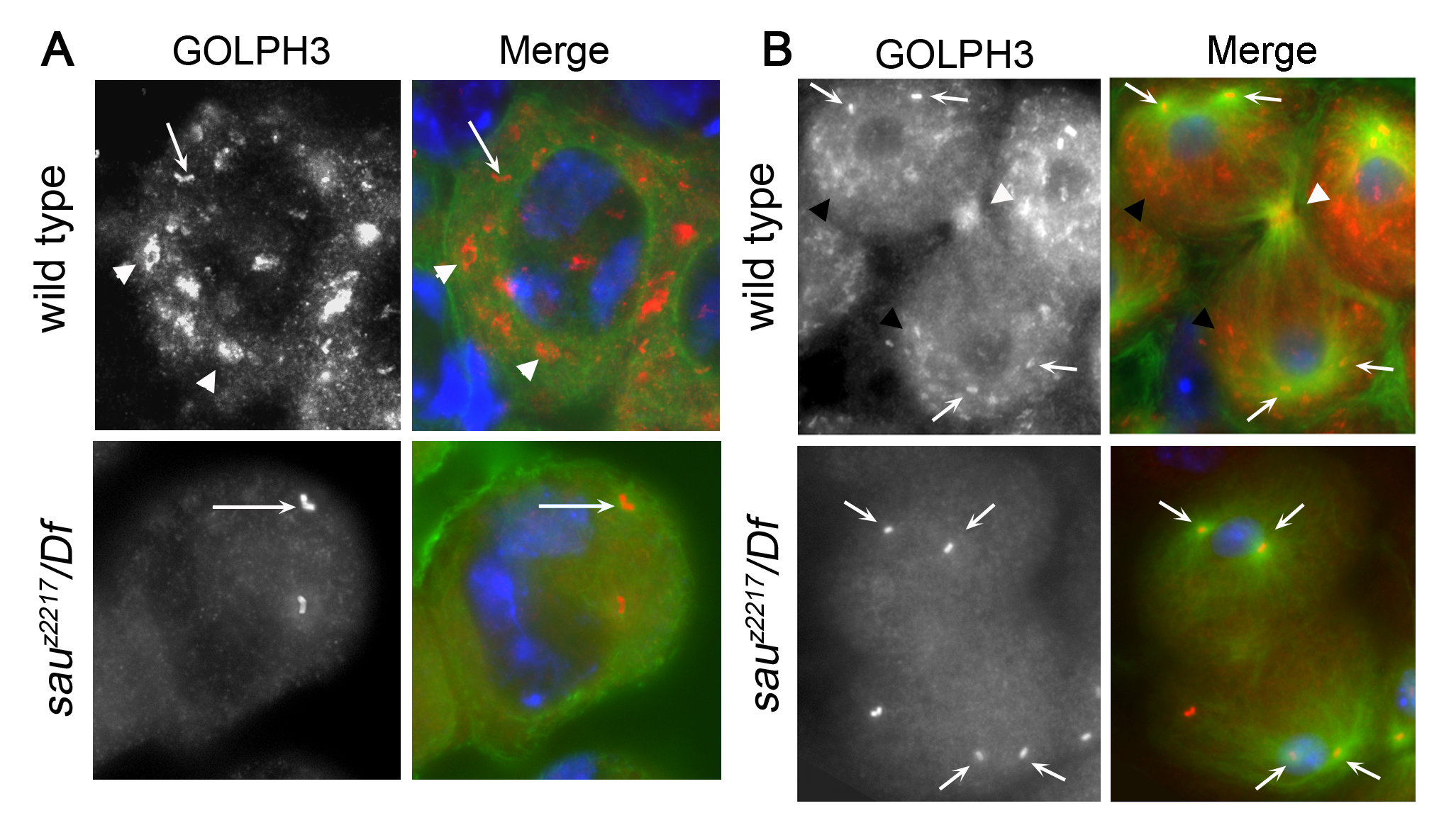

Supplement: Figure S1 — GOLPH3 protein fails to localize to the Golgi and to the midzone of sauz2217/Df(2L)Exel7010 mutant spermatocytes. Wild type and sauz2217/Df(2L)Exel7010 (sauz2217/Df) mutant spermatocytes during interphase (A) and telophase (B) stained for GOLPH3 (red), tubulin (green) and DNA (blue). White arrowheads in A point to Golgi stacks. White arrowhead in B indicates accumulation of GOLPH3 at the cleavage furrow of wild type telophase. Black arrowheads in B point to GOLPH3-enriched vesicles at the poles of wild type cells. Centriole staining (white arrows) by anti-GOLPH3 is not specific. Scale Bar, 10 µm. (TIF) [file pgen.1004305.s001.tif]

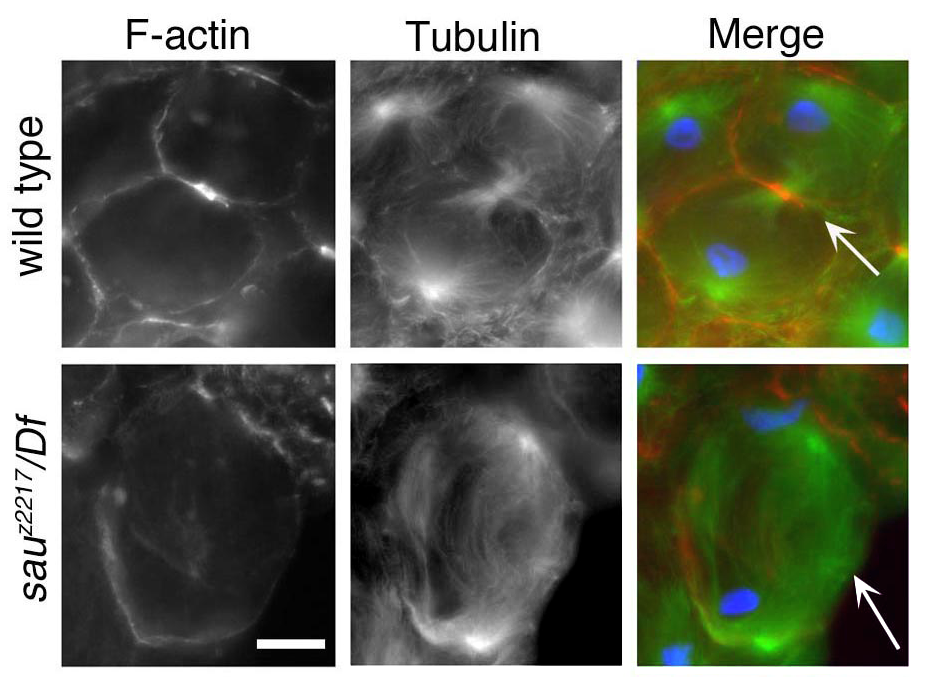

Supplement: Figure S2 — Formation of F-actin rings fails in spermatocytes from sauz2217/Df(2L)Exel7010 males. Wild type and sauz2217/Df(2L)Exel7010 (sauz2217/Df) mutant spermatocytes stained for F-actin (red), Tubulin (green) and DNA (blue) at telophase I. Arrow in wild type indicates the F-actin ring. Note the defective central spindle and the absence of a cortical F-actin ring (Arrow) in mutant cell. Scale Bar, 10 µm. (TIF) [file pgen.1004305.s002.tif]

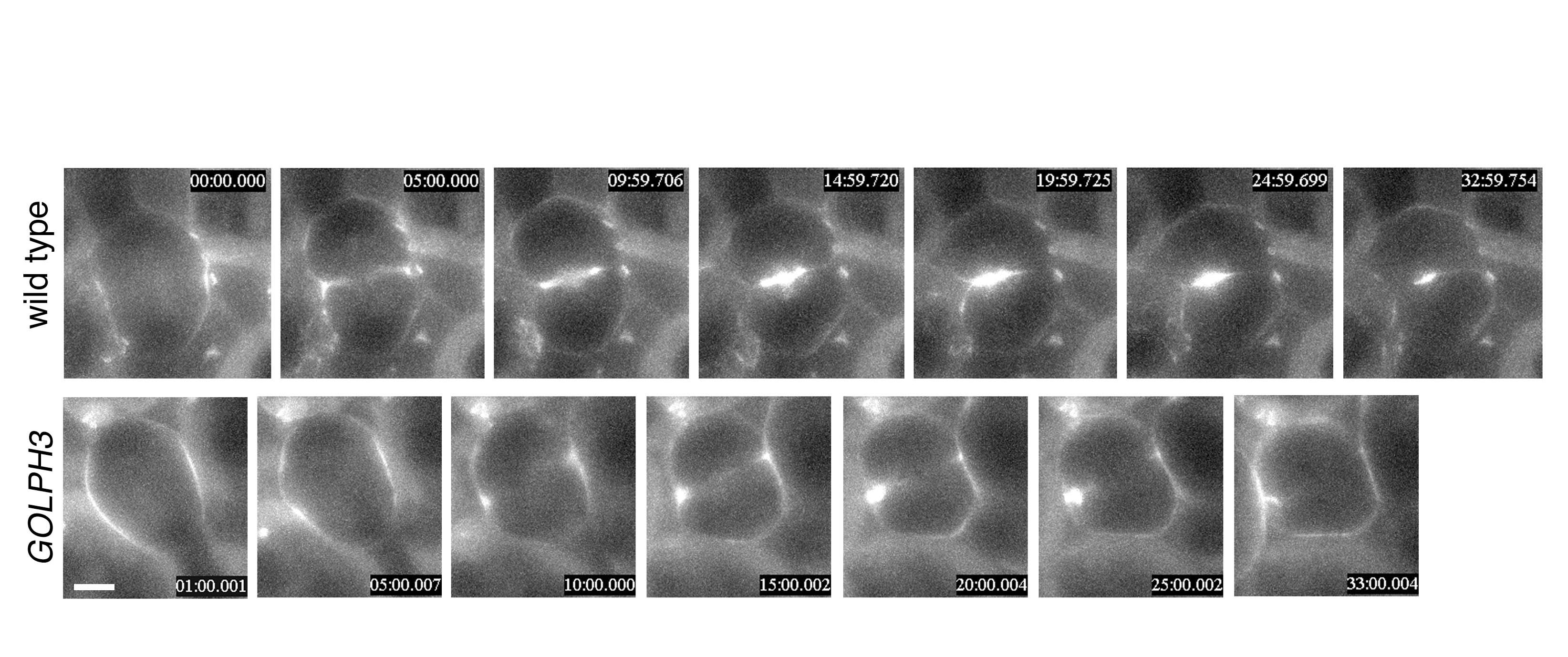

Supplement: Figure S3 — Dividing spermatocytes from sauz2217/Df(2L)Exel7010 (GOLPH3) mutant males fail to form Sqh-GFP containing rings. Frames from time-lapse sequences of spermatocytes expressing Sqh-GFP undergoing cytokinesis. Time 0 corresponds to the earliest detection of Sqh-GFP at the cell equator. Scale Bar, 10 µm. (TIF) [file pgen.1004305.s003.tif]

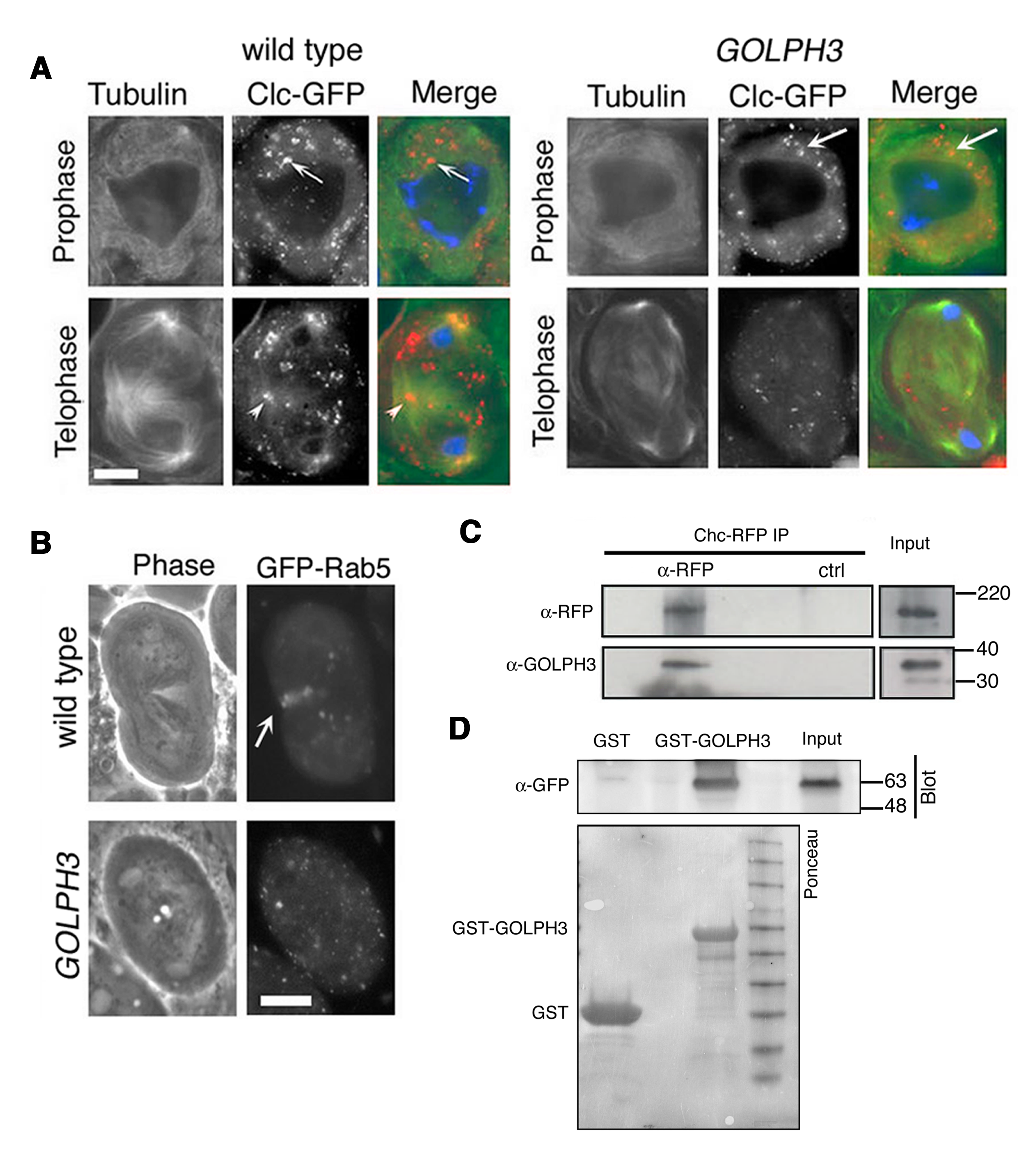

Supplement: Figure S4 — Mutations in GOLPH3 impair recruitment of endocytic markers to the cleavage furrow. (A) GOLPH3 mutations affected concentration of Clathrin at the cleavage furrow. Spermatocytes from wild type and sauz2217/Df(2L)Exel7010 (GOLPH3) males expressing Clc-GFP, fixed and stained for Tubulin (green) and DNA (blue), and GFP (GFP-Booster, red). Arrows indicate clusters of vesicular structures in interphase spermatocytes. Arrowhead indicates accumulation of Clc-GFP at the midzone of wild type telophase. Note that in mutant telophase, Clc-GFP-containing organelles appear tiny and scattered in the cytoplasm. Scale Bar, 10 µm. (B) Phase-contrast and corresponding fluorescence micrographs of telophase spermatocytes expressing GFP-Rab5. Arrow indicates accumulation of GFP-Rab5 at the cleavage furrow of wild type spermatocyte. Scale Bar, 10 µm. (C) GOLPH3 protein coprecipitated with Clathrin heavy chain (Chc) in testis extracts. Protein extracts from testes expressing Chc-RFP were immunoprecipitated with RFP-trap beads (α-RFP) and blotted for either RFP or GOLPH3. Control binding beads (ctrl) were used in control experiments. Input is 4% of lysates. Molecular masses are indicated in kilodaltons. (D) Bacterially expressed GST-GOLPH3 was purified by gluthatione-sepharose beads and incubated with testis lysates expressing Clathrin light chain tagged with GFP (Clc-GFP). GST bound to gluthatione-sepharose beads was used as a negative control. GST-GOLPH3 precipitated Clc-GFP from testis protein extracts. Ponceau staining (Ponceau) is shown as a loading control. Input is 4% of lysates. Molecular masses are indicated in kilodaltons. (TIF) [file pgen.1004305.s004.tif]

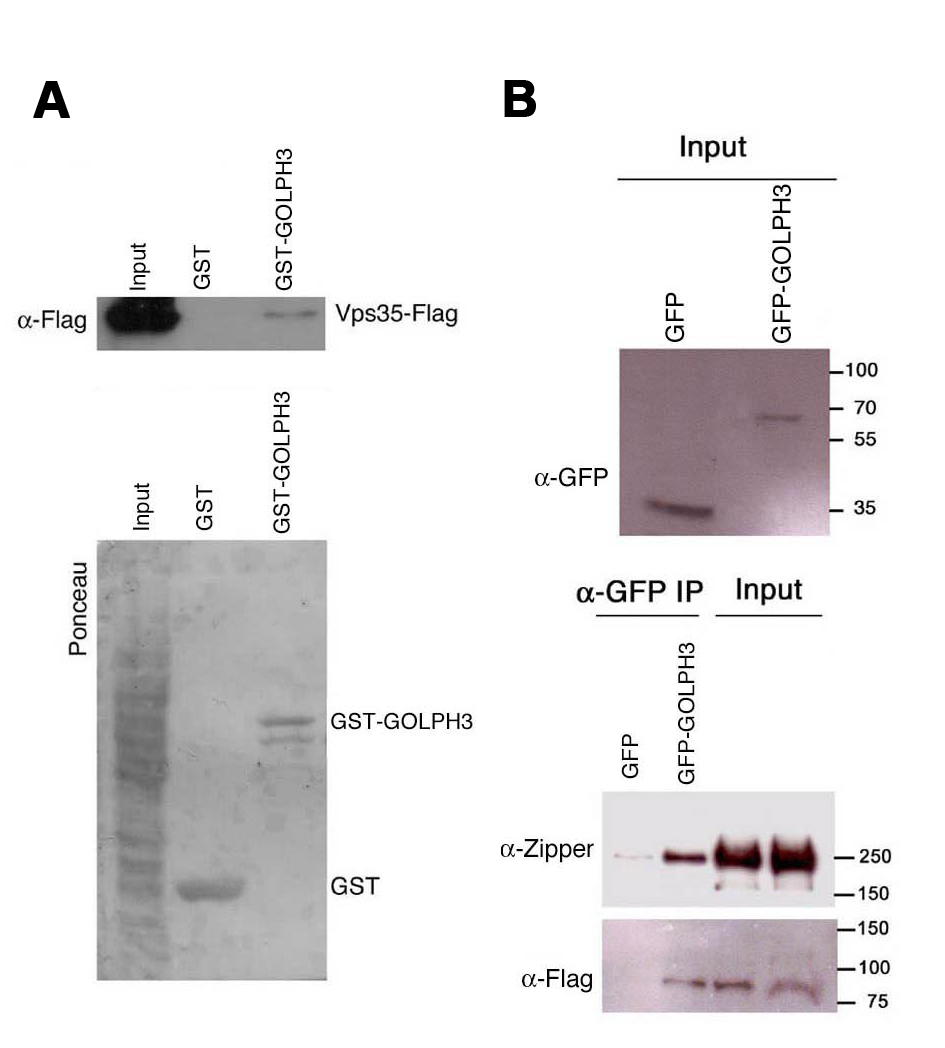

Supplement: Figure S5 — Vps35 protein coprecipitates with GOLPH3 in Drosophila S2 cells. (A) S2 cells were transiently transfected with a construct expressing Vps35-Flag. Bacterially expressed GST-GOLPH3 was purified by gluthatione-sepharose beads and incubated with S2 cells expressing Vps35-Flag. GST bound to gluthatione-sepharose beads was used as a negative control. GST-GOLPH3 precipitated Vps35-Flag from S2 cell extracts. Ponceau staining (Ponceau) is shown as a loading control. (B) Zipper and Vps35-Flag coprecipitated with GFP-GOLPH3 in Drosophila S2 cells. S2 cells were transiently transfected with a construct expressing Vps35-Flag and with either a construct expressing GFP or a construct expressing GFP-GOLPH3. Extracts from S2 cells expressing Vps-Flag and either GFP or GFP-GOLPH3 were immunoprecipitated with anti-GFP (GFP-trap) and blotted for either Zipper (α-Zipper) or Vps35-Flag (α-Flag). Western blot at the top of this panel shows the level of expression of the GFP proteins (input). (TIF) [file pgen.1004305.s005.tif]
